# Precision oncology: a review to assess interpretability in several explainable methods

**DOI:** 10.1093/bib/bbad200

**Published:** 2023-05-30

**Authors:** Marian Gimeno, Katyna Sada del Real, Angel Rubio

**Affiliations:** Departamento de Ingeniería Biomédica y Ciencias, TECNUN, Universidad de Navarra, 20009, San Sebastián, Spain; Departamento de Ingeniería Biomédica y Ciencias, TECNUN, Universidad de Navarra, 20009, San Sebastián, Spain; Departamento de Ingeniería Biomédica y Ciencias, TECNUN, Universidad de Navarra, 20009, San Sebastián, Spain; Instituto de Ciencia de los Datos e Inteligencia Artificial (DATAI), Universidad de Navarra, 31008, Pamplona, Spain

**Keywords:** interpretability, precision medicine, machine learning, explainable artificial intelligence, drug recommendation, assignment problem, method comparison

## Abstract

Great efforts have been made to develop precision medicine-based treatments using machine learning. In this field, where the goal is to provide the optimal treatment for each patient based on his/her medical history and genomic characteristics, it is not sufficient to make excellent predictions. The challenge is to understand and trust the model’s decisions while also being able to easily implement it. However, one of the issues with machine learning algorithms—particularly deep learning—is their lack of interpretability. This review compares six different machine learning methods to provide guidance for defining interpretability by focusing on accuracy, multi-omics capability, explainability and implementability. Our selection of algorithms includes tree-, regression- and kernel-based methods, which we selected for their ease of interpretation for the clinician. We also included two novel explainable methods in the comparison. No significant differences in accuracy were observed when comparing the methods, but an improvement was observed when using gene expression instead of mutational status as input for these methods. We concentrated on the current intriguing challenge: model comprehension and ease of use. Our comparison suggests that the tree-based methods are the most interpretable of those tested.

## INTRODUCTION

Precision medicine (PM) is the science that ‘defines a disease at a higher resolution by genomic and other technologies to enable more precise targeting of its subgroups’ [1]. It is an emerging field that epitomes the new era of medicine owing to its applications in clinical treatment and diagnosis [2].

PM tries to find not only the right drug for each patient but also the right dosage and the proper treatment schedule. These goals are usually summed up as ‘targeting the right treatments to the right patients at the right time’ [3]. In this review, we will focus on considering the patients’ genome to provide each patient with the ‘best’ treatment according to it. PM faces different obstacles regarding its implementation. In this review, we focused on some of them, namely, eliciting patient response to drugs, algorithmic problems and interpretability of algorithms.

Nonetheless, PM faces many more challenges, such as data quality and heterogeneity of data sources (not all the data are equally good or easy to compare) [4], scarcity of data (PM usually involves small populations treated with only one or, at most, two or three treatments) [5] and generalization (models trained on one population may not generalize well to other populations, particularly those with different demographics or genetic backgrounds) [6].

Probably, one of the most important challenges is the informed consent. This is a crucial ethical principle that involves obtaining the consent of a patient or research participant before conducting any medical intervention or study. It involves providing the participant with adequate information about the potential risks and benefits of the study, the treatment options available and any other relevant information that may help them make an informed decision.

It becomes particularly important when it comes to PM as it involves a degree of risk that the patient must be aware of before consenting to the treatment or participating in the study. A clear and comprehensible explanation of the methodology to select a treatment (‘explainable precision medicine’) makes it easier for both the patient and the physician to understand it and, henceforth, to give their consent to follow the advice of the recommended method.

### The challenge of getting the patients’ response to drugs

**Figure 1:**
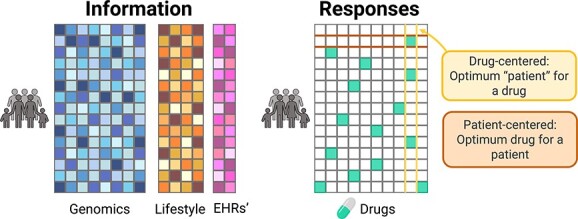
Precision medicine paradigm. The left-side panel represents patients’ data and the right-side panel shows the data available for patients’ responses to treatment.

PM requires the different patients’ characteristics to make their predictions [7] such as genomic and transcriptomic data, health records and lifestyle characteristics (Figure 1). With an adequate data policy, these characteristics are reasonably easy to obtain; genomic data can be acquired from sequencing techniques, wearable technologies can collect data that provide lifestyle information, electronic health records are invaluable sources of information on health status and previous conditions, etc. Its integrative analysis requires complex models and a solid understanding of the interaction of biological systems [8].

However, PM also requires drug sensitivity information, which is much more difficult to find, having most likely incomplete information on patients’ response to all available drugs, i.e. each patient is given one or, at most, a few drugs, not all the possible ones [9] (Figure 1). Even in these cases, distinguishing between responders and non-responders is not an easy task and requires tailoring methods specific to each disease. In turn, these different criteria for different diseases make it difficult to compare diseases or drugs [10].

The first possibility to estimate drug response is to use cell lines as a model of the disease. Different studies include prediction of drug response (using IC_50_, area under dose–response or other variants) using different -omics data [11–14]. One interesting attempt is DrugCell [15], which uses a deep neural network that mimics the structure of the gene ontology to provide insight on the predictions of the network. DrugCell as well as other machine learning (ML) methods are compared and discussed in the reference [16].

Cell lines are not reliable models of the disease. An additional step in the modeling of the disease is using patient-derived xenografts (PDXs). PDXs or *ex vivo* experiments can be used as proxies to estimate the patients’ response to several drugs [17]. Both approaches have strong limitations. Due to inherent differences in biology and physiology between humans and animals, PDXs are limited in their ability to fully reproduce the complexity of human disease. Consequently, PDXs may not accurately capture all variations observed in humans [18]. In addition, due to the large variability observed in human disease and the limited sample sizes typically used in PDX studies, it may be difficult to achieve statistical significance in studies regarding these models. Moreover, the immune system of the animals is often compromised [19].

In the case of *ex vivo* experiments—used mainly in hematologic oncology—the interaction of the cells and the immune system is not properly modeled. Despite these difficulties, there are reasonable sources of information to predict the response of the patients to different treatments [19].

Even once the data have been gathered, there is still much to be developed: the necessary quality controls must be carried out, the batch effect of the different laboratories where the data were collected must be eliminated or at least minimized, algorithms and data must be standardized to ensure that they can be combined, and so on. From this point of view, mutations, and to a lesser extent chromosomal abnormalities, are easier to share among different laboratories or technologies. In contrast, other -omics are much more difficult, if not impossible, to integrate. For example, comparing gene expression results from different laboratories using different technologies (e.g. RNAseq and microarrays) is problematic.

### PM falls beyond traditional ML problems

PM can be considered an assignment problem: each patient must be provided a drug (or a set of drugs) given the patient’s information. This assignment problem does not perfectly fit in any of the ‘traditional’ fields of ML. It is not an unsupervised problem, although, with a proper selection of variables, patients with identical ‘optimal’ drugs should cluster together [20, 21].

Regarding supervised ML, it is not either a standard regression problem since the aim is not to predict the effectiveness of a drug on each patient but to find *the most effective ones* [22]. Nevertheless, both problems are related, and, if the effectiveness of each drug were exactly modeled, the ‘perfect’ drug for a patient would be simply the most effective one predicted by the model. It could also be treated as a classification problem dividing the drugs for each patient into two classes: the most effective one belongs to one class and the others belong to another class. Again, it solves the problem if the predictions were perfect. However, since this simplistic model only considers misclassifications (the second-best drug is as bad as the worst), it does not work well in practice.

Finally, it can also be considered a reinforced learning problem [23]. For example, [24] includes a review of reinforcement learning applications to oncology. The objective of this field of ML is to learn an optimal, or nearly optimal, policy that maximizes the ‘reward function’—in this case, the patient’s response to treatment. Reinforced learning is traditionally applied to teach the computer how to play games (chess, Go or video games) [21]. Applied to PM, different methods state how to use a reinforced learning algorithm to ‘find a policy that maximizes the patient response to treatment’.

**Figure 2:**
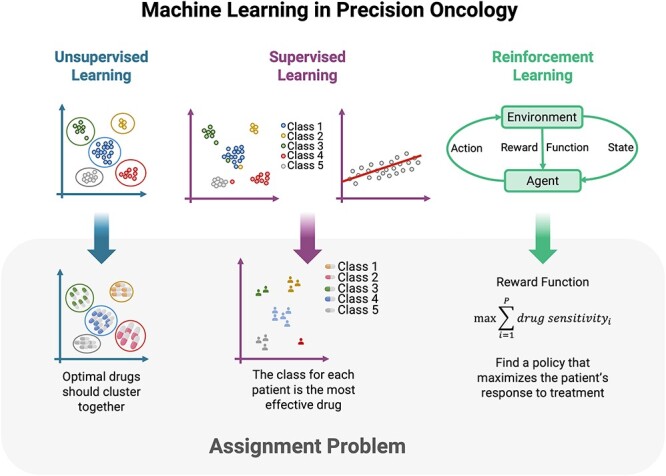
Relationship between ML and the assignment problem. The assignment problem is not a specific ML problem but could be addressed from all the ML branches.

As a result, PM—assigning the proper drug to each patient—given the patients’ data is a problem that shares characteristics of different ML fields (Figure 2) and can be tackled in many ways as will be shown in the different analyzed approaches.

### Patient-centered versus drug-centered

There are two main approaches to solving the goal of ‘targeting the right treatments to the right patients’ (Figure 1). The first one is to state which is the proper drug for a specific patient. We will name this approach ‘patient-centered’. The other approach consists of finding the patient or patients that are responders for a specific drug, named ‘drug-centered’ in this review. This problem—closely related to finding biomarkers of response—is interesting for the pharma industry.

If the output of the algorithm is a continuous value, it is possible to adopt a drug-based method to solve the patient-based problem and vice versa. For example, many drug-centered methods return a sensitivity score for each patient when applied to a specific drug. If this score is computed for all the drugs, it can be used to select the drug that maximizes sensitivity for each patient.

### The challenge of interpretability

One common problem in ML is the (lack of) interpretability. In many cases, the algorithm is a black box that gives no clues on why a specific decision is taken [25–37]. It is difficult, if not reckless, for a physician to use a treatment guideline with no information on the ultimate reasons that drove this recommendation. Explainable artificial intelligence (XAI) is an active field of research: it justifies the response and ensures that, given the a priori knowledge, the recommendation is sensible. XAI also helps to improve the results since, as they are understandable by physicians, they can provide expert feedback to fine-tune the algorithms [38–41]. Some methods have tried to explain their reasoning to become more explainable [39, 42–48].

Treatment guidelines that require few biomarkers are easier to understand by a human. Therefore, the number of variables is one of the characteristics used to determine how explainable a method is. Some methods [7, 32–36, 44, 49] automatically select the optimal number of variables to accomplish a task. In other cases, the selection of variables must be done beforehand using either a filter or a wrapper technique depending on whether the result of the predictions is included in the loop to select the variables [37, 50–53].

### Method of comparison

In this review, we included interpretable methods amenable to the PM problem, i.e. find the best drug for a patient. We systematically reviewed the current literature to summarize the state-of-the-art and compare different methods that solve the assignment problem.

Some of the methods available in the literature—that are said to be interpretable and output a drug assignment—refer to methods that are currently under the ‘patient-centered’ approach [54, 55], and others are nested under the ‘drug-centered’ approach [56–58]. From the latter, only the methods that predict a continuous variable for drug sensitivity could be used for patient assignment.

We compared the methods in this work in terms of interpretability, focusing especially on the accuracy, multi-omics capacities and translation into clinical practice. Method comparison was performed using BeatAML2 [59] dataset and Genomics of Drug Sensitivity in Cancer (GDSC) [60] dataset for acute myeloid leukemia (AML). For more details on the samples used, see Supplementary Table 1. We focused on the BeatAML2 dataset due to its richness in patient information, e.g. genomic data, gene expression and clinical data, and drug sensitivity information which proceeded from *ex vivo* experiments performed on patient samples instead of cell lines [59]. Indeed, *ex vivo* drug sensitivity provided more information for patient sensitivity than conventional information from clinical data due to the possibility of testing more drugs on the living tumor without injuring the patient and solving possible harmful drug interactions from previous treatments. Using this information—although it could be less reliable—solves the sparsity issue of drug sensitivity data. Furthermore, drug screens performed on *ex vivo* experiments improve data reliability if compared to cell line screenings. Nevertheless, further experimental validation is required for clinical applications.

AML is a blood tumor that originates in the bone marrow of the patients and has a very poor progression-free survival. In addition, it is a highly heterogeneous disease for which finding effective treatments is a challenge [40, 61–63]. Due to the technical difficulties in finding suitable data from this disease to implement a common ML model, the need to find therapeutic strategies and the availability of *ex vivo* drug screening experiments, we believe that the BeatAML2 dataset is perfectly suited for this comparison.

## METHODS

Focusing on approaches to address the complex PM problem, we found two methodologies from the ‘patient-centered’ perspective, Multidimensional Optimization Module (MOM) [54] and Kernelized Rank Learning (KRL) [55]. MOM uses mixed integer linear programming (MILP) to discover the optimal therapeutic strategy that is returned as a decision tree. KRL is a ML method based on an optimization problem, minimizing the sensitivity error; it applies a kernel to solve the convexity limitations and solves the problem also using MILP.

We also included in this group two novel algorithms: Optimal Decision Trees (ODT) and an adaptation of the Multinomial Lasso. In this review, we discuss algorithms suitable for PM that can be understood by both the physician and the patient. Artificial neural networks (including deep learning) or ensemble methods (including bagging and boosting) are difficult to understand. On the other hand, it is desirable for the algorithm to select and use only a few variables. These requirements leave only a few methods available: decision trees and regression methods. Neither decision trees nor the available regression methods fit the problem of PM. Therefore, we adapted both to solve it. ODTs are decision trees that recursively optimize the drug recommendation on each branch until a preset group size is reached. On the other hand, the Lasso-regularized multinomial regression uses a vote splitting scheme to assign the best drug to each patient. ODT and Multinomial Lasso are reasonably easy to understand and apply. They are described in more detail below.

In the ‘drug-centered’ approach, BOSO [56] and Lasso Regression [58] can be applied to predict the response of a drug in different patients. Both methods select a small number of variables to make their predictions. Once the predictions are obtained, the predicted response for each drug in a patient is compared and the drug with the optimal response is selected. BOSO is a MILP model built up from the Lasso Regression equations that have been modified to predict a numeric variable with the least number of features, improving the reduced interpretability of Lasso Regression. The description of the six methods is summarized in Table 1.

**Table 1:** Precision medicine pipelines selected for comparison

Algorithm	Type	Software	Method	Suitable for mutational data	Suitable for gene expression	Output	Reference
MOM	Patient	Python 3.7, R 4.2 and CPLEX	Feature selection and MILP	Yes	No	Drug assignation	[45]
ODT	Patient	R 4.2	Recursive Decision Tree	Yes	Yes	Drug assignation	Novel
Multinomial	Patient	R 4.2	Multinomial Lasso	Yes	Yes	Drug assignation	Novel
KRL	Patient	Python 2.7	Kernelized MILP	Yes	No	Drug assignation	[54]
BOSO	Drug	R 4.2 and CPLEX	Lasso regression using MILP	Yes	Yes	Predicted IC_50_ for a drug	[47]
Lasso	Drug	R 4.2	Standard Lasso regression	Yes	Yes	Predicted IC_50_ for a drug	[7]

This table collects the description of each of the methods. Algorithm shows the method’s given name. Type refers to whether the method is patient- or drug-centered. The software column collects all the required software environment programs for the model to be run. Method refers to the pipeline description. Suitable for mutational data has a ‘Yes’ if the method could use genetic variants as input. Suitable for gene expression has a ‘Yes’ if the method could use gene expression data as input and a ‘No’ otherwise. Output explains the raw output of the model. Reference contains the publications in which the method was defined.

KRL and MOM only accept binary inputs. The other methods (ODT, Lasso, Multinomial Lasso and BOSO) accept both binary and continuous inputs. The first two methods were tested only using mutational data input. The other methods were tested using mutational data and/or expression data.

### Optimal Decision Trees

In this work, we are introducing a novel algorithm that uses a tree-like method for PM. This method is intrinsically different from classification or regression trees, as will be shown.

In a classification tree, in each step, the tree is split into two subtrees finding the variable (with its corresponding threshold) that best splits the tree according to some figure of merit (Gini index, entropy, information gain, etc.). This figure of merit measures the overall enrichment of the classes in the subtrees.

**Figure 3:**
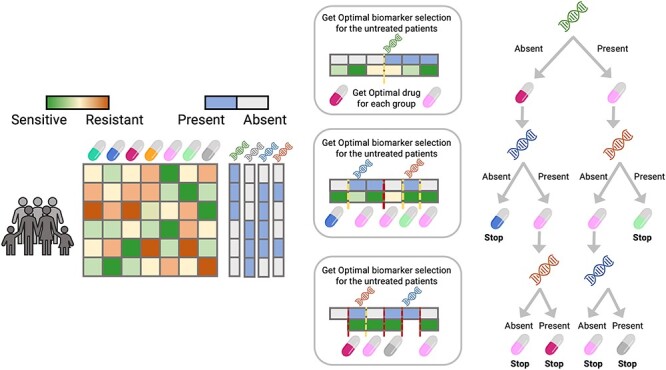
ODT model performance. The ODT model uses as input the sensitivity matrix and the biomarker matrix, on each step it splits the patients into two groups according to the presence or absence of a biomarker. This split is optimized so that the drug-assigned is the most sensitive to each of the splits. It recursively splits the different branches until a predefined group size is reached.

On the contrary, the ODT algorithm selects for each step the splitting variable (selecting a proper threshold) *and the treatments for each split*. The selection is based on the optimization of an overall measure of the sensitivity of both branches to the selected treatments (Figure 3).

Specifically, let Y be a P × D matrix where P is the number of patients and D is the number of tested drugs. Each of the entries of the matrix quantifies the sensitivity of each patient to a drug, i.e. the matrix Y can be either the IC_50_ or a modified version of it, the area under the concentration–response curve, etc. Let X be a P × M matrix where P is the number of patients and M is the number of biomarkers. The matrix X can be a matrix of mutations, gene expression or other characteristics specific to each patient.

In the case of binary variables (mutations for example), for each step in the splits of the tree, the following optimization problem is solved (Equations (1)–(3)):


(1) \begin{equation*} \underset{m,{d}_1,{d}_2}{\max}\ A+B \end{equation*}

(2) \begin{equation*} A=\sum_{p\in split}{y}_{p{d}_1}\left({x}_{pm}==1\right) \end{equation*}

(3) \begin{equation*} B=\sum_{p\in split}{y}_{p{d}_2}\left({x}_{pm}==0\right) \end{equation*}

where split is the set of patients under study (all patients are used in the case of the root node), *m* is the selected mutation or biomarker, *d*_1_ and *d*_2_ are the selected drugs for the patients that have or do not have the mutation *m*, respectively, and *A* and *B* are the sum of the measured responses for treatments *d*_1_ and *d*_2_, respectively. The notation ‘(*condition*)’ represents 1 or 0 depending on whether the expression inside the parenthesis is true or false (Equations (2)–(3)). This problem can be easily extended to continuous variables, using a threshold (Equations (4)–(6)). In this case, the optimization problem is


(4) \begin{equation*} \underset{m, th,{d}_1,{d}_2}{\max }A+B \end{equation*}

(5) \begin{equation*} A=\sum_{p\in split}{y}_{p{d}_1}\left({x}_{pm}>= th\right) \end{equation*}

(6) \begin{equation*} B=\sum_{p\in split}{y}_{p{d}_2}\left({x}_{pm}< th\right) \end{equation*}

Both optimization problems start by setting all the patients within an initial group. The optimization splits the patients into two groups. For each of these groups, the algorithm is applied recursively until the number of patients in the split is smaller than a given number or until the optimization problem results in the same drug for both splits.

Equations (2)–(6) maximize the sum of the sensitivities of the patients of each of the branches. Using the same algorithm, it is possible to use any transformation of the sensitivity and include them in the optimization process. In this case, Equations (5) and (6) are transformed into


(7) \begin{equation*} A=\sum_{p\in split}f\left({y}_{p{d}_1}\right)\left({x}_{pm}\ge th\right)\ B=\sum_{p\in split}f\left({y}_{p{d}_2}\right)\left({x}_{pm}< th\right) \end{equation*}

Equations (2) and (3) can be transformed analogously. To minimize the effect of outliers in the sum, we used the square root function to diminish the dynamic range of the data. The transformation is named ODT Sqrt in this work.

### Multinomial logistic Lasso regression

The assignment of the proper drug to each patient problem can be tackled as a multiclass classification problem: the number of classes is the number of drugs, and each patient is assigned the most effective drug for him/her. Using this approach, a multinomial regression can be applied to select the proper drug for each patient.

Predicting exclusively the most effective drug can be simplistic since the penalty for misclassification is identical for the second most effective drug or for the least effective drug. Since the multinomial regression can also be applied to continuous variables, it is possible to give a ‘vote’ for each patient that can be shared among all the drugs: the most effective drug will receive more shares of this vote than the least effective drug. Assigning the whole vote to the most effective drug can be seen as a particular case of this approach.

The Lasso penalty is also implemented for multinomial regression. The implementation of glmnet (R Package) [58] is fast and convenient and allows for the automatic selection of the regularization parameters using cross-validation.

More specifically, the multinomial regression builds the multinomial regression model (Equation (8))


**Figure 4:**
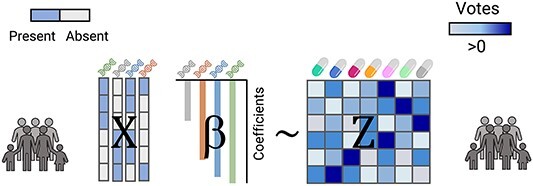
Multinomial model. The multinomial model corresponds to a modified multinomial logistic Lasso regression, where the output represents the votes that each patient assigns to each of the drugs.

(8) \begin{equation*} \boldsymbol{X}\boldsymbol{\beta } \sim \boldsymbol{Z} \end{equation*}

- where ***X*** is a P × M matrix where P is the number of patients and M is the number of biomarkers, and $\boldsymbol{Z}$ is a P × D voting matrix (in fact, probabilistic labels) where P is the number of patients and D is the number of tested drugs. All the elements of ***Z*** are positive and the sum of its elements by rows is equal to one. Finally, $\boldsymbol{\beta}$, the output of the regression, is a M × D coefficient matrix. $\boldsymbol{X}\boldsymbol{\beta }$ are the predicted logits for each drug being the most effective for each patient (Figure 4).

The specific selection of the entries for the ***Z*** matrix is shown in Equation (9):


(9) \begin{equation*} {z}_{pd}=\frac{\exp \left(-K\frac{y_{pd}}{\underset{p}{\min}\left({y}_{pd}\right)}\right)}{\sum_{i=1}^D\exp \left(-K\frac{y_{pi}}{\underset{p}{\min}\left({y}_{pi}\right)}\right)} \end{equation*}

where *y_pd_* are the entries of the Y matrix (that measures the sensitivity to a drug) and *K* is a predefined constant. If *K* >> 1, all the exponentials of the summations of the denominator except the *y_pi_* that corresponds to min(*y_pi_*) vanish and the vote is given to the most effective drug. If *K* = 0, all the drugs share 1/D votes.

More information regarding the other algorithms already published can be found in the Supplementary Material (see section Supplementary Methods).

### Data for comparisons

We focused on AML to compare the different methods described above. This disease was selected due to the availability of a wide cohort of patients with genomics data and *ex vivo* drug sensitivity screening data. *Ex vivo* data are more reliable than drug screenings performed on cell lines since the tests are performed directly on the AML patients’ blood. Furthermore, AML is a highly heterogeneous disease with no standard PM therapeutic strategy, even though there is a growing field of drug development likely suited for these patients, e.g. tyrosine kinase inhibitors [64]. In addition, there is a list of FDA recently approved drugs for the treatment of AML, such as Daurismo (glasdegib) or Venclexta (venetoclax) [65].

Accordingly, we selected Waves 1 + 2 from the BeatAML2 cohort [49] to train the models and Waves 3 + 4, created later, to test the predictions of the different therapeutic strategies. This dataset is publicly available at https://biodev.github.io/BeatAML2/.

Instead of using the standard IC_50_ from the *ex vivo* experiments, we propose to use an incremental version of the IC_50_ called IC_50_^*^, described in more detail [54]. The only difference between the IC_50_ and the IC_50_^*^ is that in the latter, the logarithms of the IC_50_ are subtracted the average value of the logarithm of IC_50_ for each drug, so that the mean of the logarithms of the IC_50_^*^ of each drug is zero. This correction prioritizes drugs that have a differential effect in different patients, which are, in turn, better candidates for developing a personalized treatment based on a companion biomarker. In addition, changes in drug potency can be counterbalanced by appropriate dose selection.

To validate the predictions, we also used as an independent cohort testing set the GDSC drug screening for AML cell lines [60], which is publicly available at https://www.cancerrxgene.org/.

**Figure 5:**
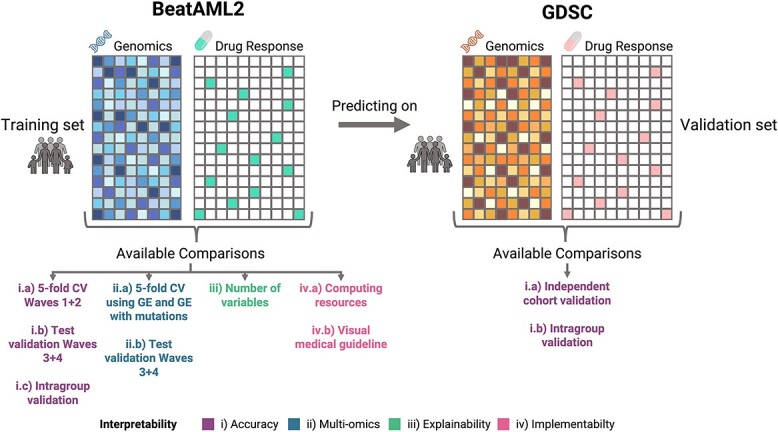
Summary of the available comparisons performed in this study. We trained the different models in BeatAML2 Waves 1 + 2 cohort and tested the predictions predicting over GDSC. From the training step, we were able to obtain the wall clock training time, the number of variables required to make the predictions, three 5-fold cross-validation using mutational, gene expression data, mutations together with gene expression data and an intragroup validation, whereas for the testing step, we performed and independent cohort prediction validation using mutational data, and another intragroup validation.

We compared the different algorithms based on four aspects that define interpretability (Figure 5): (i) *the accuracy* of the method, for which we performed a 5-fold cross-validation in the training set, a test validation (in Waves 3 + 4 of BeatAML2), an independent validation (with GDSC) and an intragroup validation with the predicted groups in the test and independent cohort set; (ii) *the multi-omics capacity*, for which we tested the ability and performance of the methods when training with gene expression and gene expression together with genomic variants; (iii) *the explainability*, for which we performed a qualitative comparison of all algorithms, analyzed the number of variables that each algorithm uses for prediction; and (iv) *the implementability*, for which, apart from qualitative comparisons based on the definition of the method, the computation time required for training each model and the possibility of having a graphical interface become essential.

These four categories are explained more in depth in the following paragraphs.

#### Accuracy

The first ‘*sine qua non*’ characteristic of PM methods is accuracy. An ‘interpretable’ method with low accuracy becomes irrelevant. We define accuracy as the difference between the IC_50_^*^ for the assigned drug and the drug with the minimum IC_50_^*^ for that patient.

For assessing the accuracy of each of the methods, we performed the following comparisons: 5-fold cross-validation, test validation, independent cohort validation and intragroup validation.

**Figure 6:**
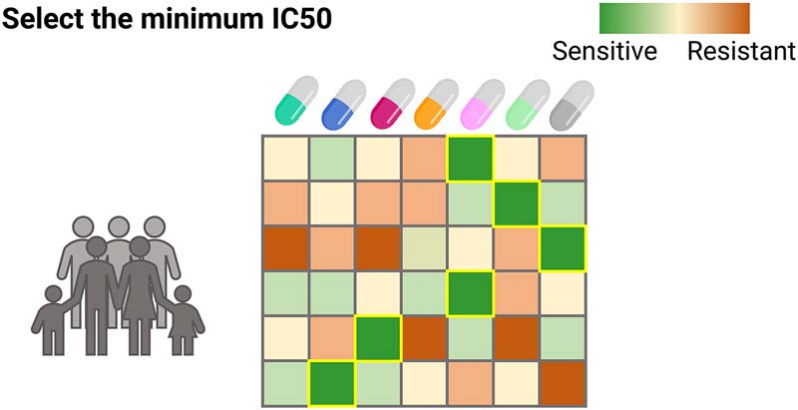
Oracle prediction. The Oracle predicts the most sensitive drug for each patient or cell line.

*Fivefold cross-validation in BeatAML2*. We performed a 5-fold cross-validation using the BeatAML2 dataset. We trained all models with genetic variants data from 247 patients belonging to Waves 1 + 2 of the dataset, dividing the cohort of training samples into 5-folds; four were used to train and the remaining one was used for testing. The role of the test fold is changed in every training, so that each of the folds was tested. The predicted IC_50_^*^ for the 5-fold testing was compared for all the methods and compared against the Oracle—the drug with the minimum IC_50_^*^ (Figure 6). Then, we tested the final models trained on the 247 patients of the Waves 1 + 2 using 142 patients of the Waves 3 + 4 of BeatAML2.

*Independent cohort validation*. One of the main challenges of ML, including PM, is the generalization, i.e. the ability to adapt to new, previously unseen data. All the methods were tested on the GDSC AML dataset to check their generalization ability. The models were trained using the BeatAML2 dataset and were used to predict the optimal drug for AML cell lines from GDSC using its mutation files. Each of the cell lines was recommended a drug, and we compared the all-sample IC_50_ for all the models and against the Oracle (the drug with the minimum IC_50_^*^ for each cell line).

*Intragroup validation*. We compared whether the ∆IC_50_^*^ of a drug in patients in whom it was recommended was lower than the ∆IC_50_^*^ in patients in whom it was not recommended. The ∆IC_50_^*^ is simply the difference between the IC_50_^*^ obtained and the one of the Oracle. Using this information, we compared the sensitivity to a drug for a specific group against the sensitivity to that drug for the rest of the samples by using a two-tailed Wilcoxon test. This analysis was performed for both validation datasets, the BeatAML2 Waves 3 + 4 cohort and the GDSC AML cell lines cohort.

#### Multi-omics suitability

Some of the methods only accept as input binary variables. Although genomic variants can be transformed into binary variables, gene expression, methylation or openness of the chromatin are intrinsically continuous. We have included a table showing whether the algorithm accepts only binary inputs (genomic variants only) or it also accepts continuous data (gene expression, methylation, etc.). For the methods that accept continuous variables (all methods except MOM and KRL), we assessed the performance of the predictions (5-fold cross-validation) in the BeatAML2 dataset using genomic variants, gene expression and a combination of both. We state the statistical significance using a two-tailed Wilcoxon’s test comparing the IC_50_^*^ using as input genetic variants, gene expression or both.

#### Explainability

PM is more suited for healthcare if it can be interpreted. An ML method is interpretable if it provides the decision criteria that define the pathway that leads to the solution.

Explainability is defined by three different aspects: (i) the explainability of the results, which checks if the method provides a ranking of the variables according to their importance for drug recommendation; (ii) the capacity to output easy-to-apply decision criteria; (iii) the understandability of the methods—this category mentions if the process of the algorithm to reach the classification criteria is easy to understand.

For assessing these characteristics, we performed a qualitative analysis based on the method description and execution. Furthermore, we analyzed the number of variables that each model requires to make the predictions. A model with a reduced number of variables is easier to understand, improves the understanding of the variable ranking and facilitates clinical diagnosis. Therefore, we paid special attention to the number of variables.

#### Implementability

Implementability is the easiness of a method being implemented into clinical research or practice. We measured the implementability of a method by analyzing five main features: (i) the feasibility for wet-laboratory validations, (ii) the consideration of the physician’s experience, (iii) the generation of a clinical guideline, (iv) the possibility of having a graphical interface and (v) technical implementation, which refers to the computational burden and software that the method requires. We used qualitative grades for the first characteristics. Regarding the technical implementation, we considered the computational burden. Although it could be considered less important, some of the algorithms require a lot of training time. By requiring fewer resources, an algorithm is more attractive for application to larger data sets. We also analyzed the software environment that each model requires to be run.

## RESULTS

In this work, we compare several aspects of the performance of different interpretable models [66]. These models were classified into two main groups. The first one, named patient-based, are models that return a specific therapeutic strategy for each patient. The second one, named drug-based, are models that provide the patient(s) that are especially sensitive to a specific drug. Patient-based models include MOM [54], ODT, KRL [55] and Multinomial Lasso. Drug-based models are more suited for physicians and clinical investigation. This group comprises BOSO [56] and Lasso [58]. Patient-based methods rank the effectiveness of the drugs for a specific patient. Drug-based methods rank the effectiveness of a specific drug for each of the patients.

All the methods were developed to predict the drug response or develop a treatment strategy using genetic variants information. Thus, we trained the methods to predict drug efficacy using patients’ samples and *ex vivo* drug efficacy from the BeatAML2 [59] dataset. The methods were compared in terms of interpretability, which was defined according to four properties, namely, accuracy, adaptability, explainability and easiness of implementation.

### Accuracy: all the methods provided good estimates

**Figure 7:**
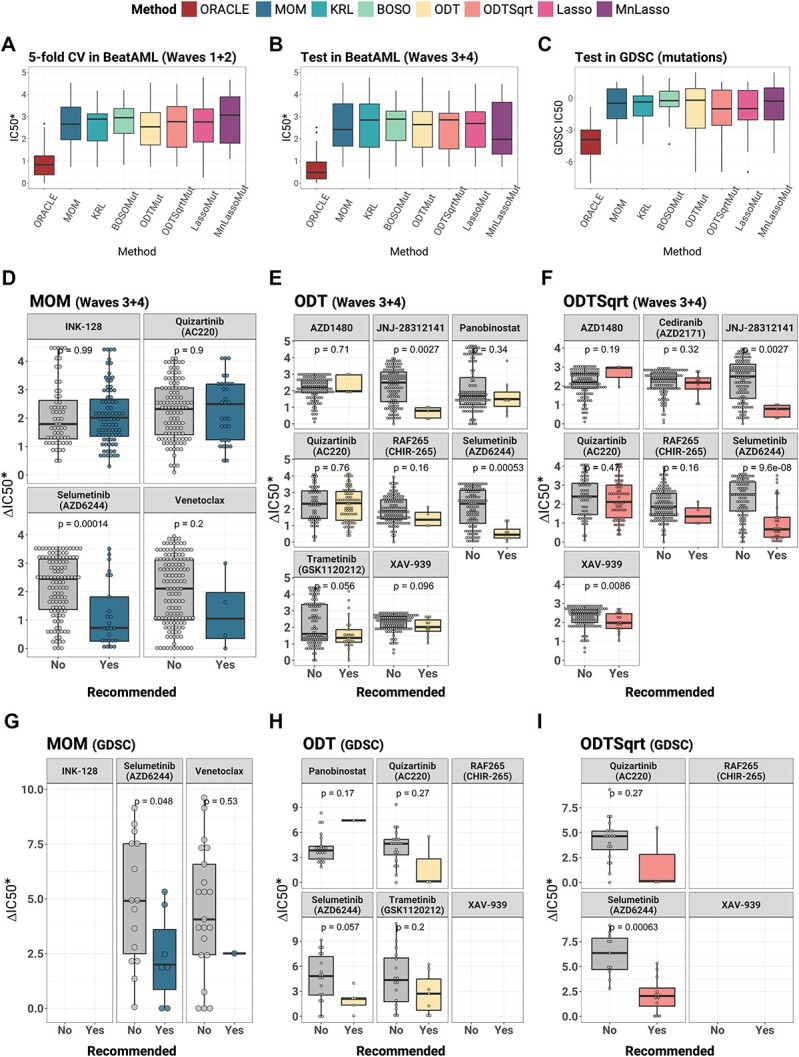
Accuracy comparison. (**A**) Accuracy in 5-fold cross-validation from BeatAML2 Waves 1 + 2 cohort. The different boxplots show the predicted IC_50_^*^ of the drugs assigned to each of the patients. The lower the IC_50_^*^ is, the more sensitive the method is, ORACLE is the control that shows the best possible drug to every patient in the cohort. (**B**) Accuracy when predicting in the BeatAML2 Waves 3 + 4 cohort. (**C**) Accuracy in independent cohort validation. The different boxplots show the predicted IC_50_ of the drugs assigned to each of the patients in GDSC. The lower the IC_50_ is, the more sensitive the method is, ORACLE is the control that shows the best possible drug to every patient in the cohort. Models were trained in BeatAML2 Waves 1 + 2 and predicted over GDSC. (**D**–**F**) Intragroup validation of MOM, ODT and ODT Sqrt in BeatAML2 Waves 3 + 4. Each of the subplots represents the efficacy of one drug. (**G**–**I**) Intragroup validation of MOM, ODT and ODT Sqrt in GDSC.

The first test to assess accuracy was a 5-fold cross-validation using mutations in BeatAML2 [59]. Figure 7A depicts the findings of this analysis. ODT has the lowest median, thus, the highest sensitivity values. ODT Sqrt and Lasso follow with nearly identical medians. The Multinomial Lasso has the highest median and the highest variance in its predictions.

However, in the second test, when predicting Waves 3 + 4 (Figure 7B), multinomial had the lowest median, but large differences were not found between its predictions and those of BOSO, which had the highest median. The results for both BeatAML2 cohorts are similar, as seen when comparing both the figures.

In the third test, we used the models trained on the Waves 1 + 2 of BeatAML2 and tested them against the GDSC AML dataset. Results are shown in Figure 7C. This dataset contains the genetic variants information for each cell line and the IC_50_ values for most of the drugs in the same cell lines. ODT Sqrt has the lowest median in this scenario, which is very close to Lasso. Also, ODT has the highest median, but once more, the results do not differ substantially from the best.

In the fourth test, we analyzed the intragroup classification performance. In this test, we compared the ∆IC_50_^*^ of patients for whom a drug was recommended with the ∆IC_50_^*^ for that drug of the remaining patients using BeatAML2 Waves 3 + 4 and GDSC. The comparisons for MOM, ODT and ODT Sqrt are displayed in Figure 7. As shown in Figure 7D and G, in both datasets, the use of MOM showed a significant difference in those patients for whom selumetinib was recommended. With venetoclax, although the difference is not significant, values are also lower in the recommended patients. ODT standard achieved a significant intragroup sensitivity in two out of eight groups for BeatAML2 (Figure 7E) and one out of four for GDSC (Figure 7H). Finally, ODT Sqrt significantly recommended the usage of three drugs out of seven for BeatAML2 (Figure 7F) and one out of two in GDSC (Figure 7I). It is important to note that the small number of samples in both datasets (142 for Waves 3 + 4 and 23 for GSDC) influences the significance of the results. Results for the other methods are given in Supplementary Figures 4–11.

### Accuracy: using gene expression as input provides greater accuracy compared to genetic variants

We tested whether the use of gene expression or genetic variants together with gene expression could improve the accuracy of the methods [67]. We trained all models (except MOM and KRL, as they do not accept continuous inputs) using BeatAML2 gene expression (GE) data. We performed two 5-fold cross-validations with BeatAML2 Waves 1 + 2, the first using GE as input and the second using genetic variant data together with GE. We then tested the models trained with the Waves 1 + 2 cohort using the Waves 3 + 4 cohort.

**Figure 8:**
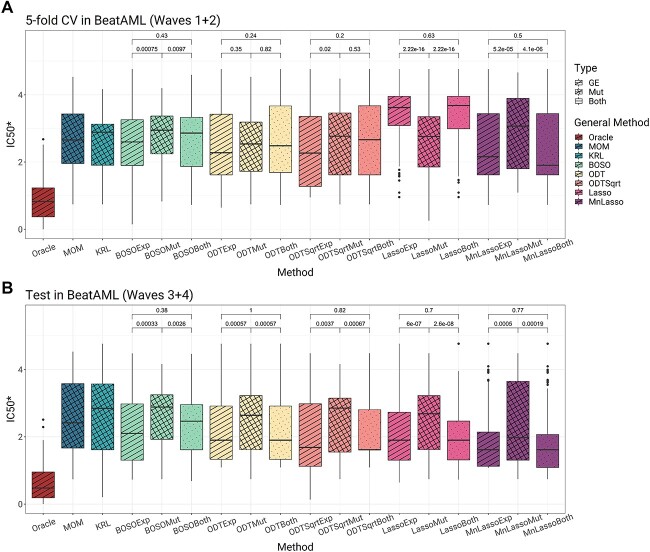
(**A**) BeatAML2 Waves 1 + 2 comparison of the different accuracies when performing a 5-fold cross-validation with all methods and using all three types of input data (when possible): mutations, gene expression and the combination of both. The ORACLE, shown in red, is the control that shows the best possible drug to every patient in the cohort. The *P*-values are shown to indicate the difference in using the different types of input data within a method. (**B**) Test accuracy when predicting with BeatAML2 Waves 3 + 4 in all methods trained on all samples of the Waves 1 + 2 cohort.

The results in Figure 8 show that the use of expression reduces the median IC_50_^*^ in almost all cases, except for Lasso using Waves 1 + 2. In fact, when testing in Waves 3 + 4, the difference between the use of GE and that of mutations was significant in all methods. Using both types of input, the median obtained is approximately similar to that of the lowest value for each method.

As shown in the preceding paragraphs, the differences between methods are minor, and no method significantly outperformed the others in all cases. However, Multinomial Lasso, followed by the ODT methods, are the ones with the lowest medians using GE and GE combined with mutations.

### Explainability: tree-like methods (MOM and ODT) require much fewer variables than any other methods

**Figure 9:**
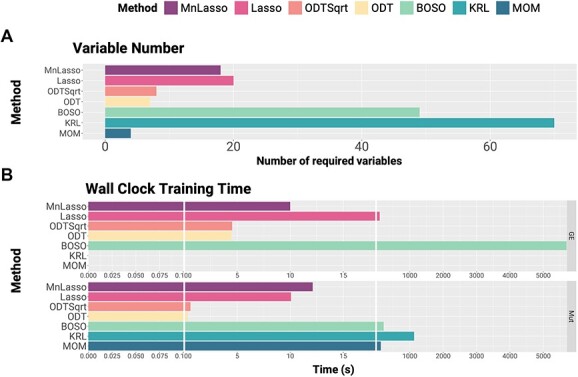
Variable number and wall clock training time comparisons. (**A**) Variable number comparisons. All methods were trained in BeatAML2 Waves 1 + 2 cohort, after the training process we extracted the number of non-zero weighted input variables that each model requires for making the predictions. The horizontal axis shows the number of variables required by each method. (**B**) Wall clock training time comparison. We measured the training time that each model requires using genetic variants (lower plot) or gene expression (upper plot) as input; time is shown in seconds in the horizontal axis.

To measure the explainability of the method, we trained the models with the BeatAML2 dataset and checked the number of variables that each model required to make the predictions. Results are included in Figure 9A**.** Remarkably, MOM and ODT use less than 10 variables. Multinomial Lasso and Lasso use 20 or fewer variables. BOSO (with 49 variables) builds a linear model for each of the drugs. The number of variables to predict each drug is small: it requires only five variables to predict drug response for some drugs. However, since these variables are not identical for every drug, in the end, BOSO requires 49 variables to make the predictions for all the drugs (Supplementary Figure 14). Multinomial Lasso and Lasso were coded to preserve the same variables for predicting all the drugs using grouped Lasso penalty (Supplementary Figures 12–13). The KRL method does not provide automatic feature selection but uses regularization methods. Therefore, all the 70 genetic variants are used.

**Figure 10:**
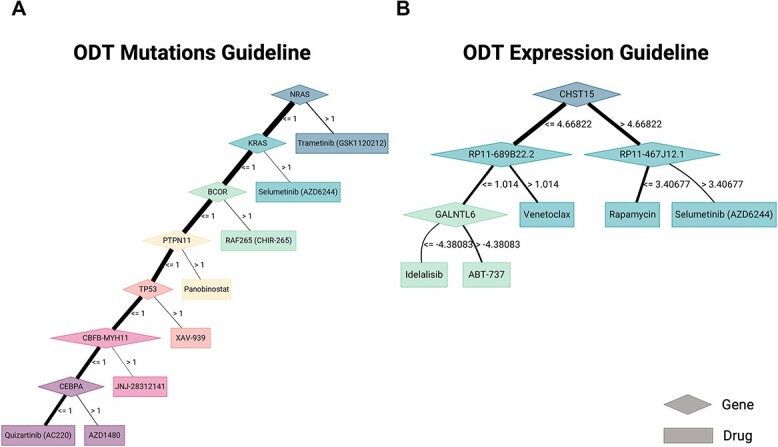
Visual representation of ODT. (**A**) Decision tree obtained after running the ODT method with mutations. (**B**) Decision tree obtained after running the ODT method with expression.

ODT and MOM output the decision criteria in the form of a decision tree. Figure 10A and B show examples of the output pattern after training ODT using mutations and GE, respectively (a similar result could be programmed for MOM). The main difference between ODT and MOM decision trees is their structure; the ODT tree may have several branches in which there are drugs for each branch as in Figure 10B. In contrast, the structure of the MOM tree is always linear, it is divided into different sequential steps, each defined by a biomarker, and there is a drug recommendation at each step. The regression-based methods (BOSO, Lasso and Multinomial Lasso) provide the weights for each of the biomarkers in each of the drugs (Supplementary Figures 12–14). Therefore, it is possible to test which biomarkers are critical for each drug. KRL uses kernels to guess the appropriate treatment. In this case, it is much more complex to understand which genomic variants are key in the recommendation system.

### Implementability: ODT and MOM are the most prone to clinical practice and ODT the least computing time consuming

We also considered the easiness to implement the methods in the wet laboratory or even clinical practice according to four different points: (i) the feasibility of wet-laboratory validations, (ii) the consideration of the physician’s experience, (iii) the generation of a clinical guideline and (iv) the computational implementation.

Tree-based models require fewer biomarkers than regression models or KRL. Furthermore, only a few operations are required to perform the predictions, which can be done by hand. On the contrary, regression models and KRL require more genes and a computer-based environment to perform the drug assignment.

As for the computational burden of each of the methods, it is necessary to train them in different software environments, R or Python. Once trained, the tree-based models directly provide a guideline that does not require the environment anymore. We have timed the training process of the six models (Figure 9B) using mutational data and gene expression (where possible). ODT is the fastest method to train (0.34 s for training using mutational data and less than 5 s using gene expression data). Multinomial requires around 12 s using either mutational data or gene expression data. Lasso lasts approximately 10 and 100 s using mutational and gene expression data, respectively. MOM took less than 3 min; however, it is important to note that increasing the number of samples results in a huge increase in training time (from minutes to hours). Using mutations as input, KRL takes the longest, approximately 19 min. Finally, BOSO requires 4 min with mutational data and with expression, which puts it at a strong disadvantage if compared to the other algorithms, taking over 1.5 h. MOM and KRL are not suitable for gene expression data, so they have been excluded from the timing analysis with this data. Prediction time is similar (and negligible if compared with training time) in all six methods. The training time when using both types of inputs is very similar to using expression. Focusing on the installation, models based on MILP (BOSO, KRL and MOM) require a complex installation of software (Table 1). They are also the most time-consuming methods. ODT, Multinomial and Lasso only require R installation to run.

All the methods could profit from a graphical interface to better understand their behavior and facilitate its use. However, ODT and MOM are especially intuitive as shown by Figure 10: both describe how to find the proper drug for each patient given a set of rules.

All these conclusions that could lead to rank methods according to interpretability have been summed up in Table 2.

**Table 2:** Table containing the interpretability comparisons for each method

Method	Multi-omics		Explainability			Implementability			
	Gene expression	Genetic variants	Small number of variables	Understandable method	Outputs decision criteria	Easy to validate	Considering experience	Clinical guideline	Computational burden
MOM	∅	^*^	^*^^*^^*^	^*^	^*^	^*^^*^^*^	^*^^*^^*^	^*^^*^^*^	^*^^*^
ODT	^*^	^*^	^*^^*^^*^	^*^	^*^	^*^^*^^*^	^*^^*^^*^	^*^^*^^*^	^*^^*^^*^
Multinomial	^*^	^*^	∅	^*^	∅	^*^	∅	∅	^*^^*^
Lasso	^*^	^*^	∅	^*^	∅	^*^	∅	∅	^*^^*^
BOSO	^*^	^*^	∅	^*^	∅	^*^	∅	∅	∅
KRL	∅	^*^	∅	^*^	∅	^*^	∅	∅	∅

The values No (Ø), Yes (^*^) and Very (^*^^*^^*^) reflect whether the method does not, fulfills or greatly fulfills, respectively, the conditions mentioned in the columns. ‘Gene expression’ refers to whether the method is suitable for this type of omics data, ‘Genetic variants’ refer to whether the method is suitable for this type of omics data. ‘Small number of variables’ refers to the number of variables that the method uses for classification, ‘Understandable method’ is defined as the logical explanation of the method; i.e. it does not rely on random selection, ‘Outputs decision criteria’ refers to whether the method provides the decision criteria used in the assignation, ‘Easy to validate’ refers to the ability of the method to be wet-laboratory validated. ‘Considering experience’ shows whether the method could enhance the physician or researcher experience. ‘Clinical guideline’ shows methods that output a therapeutic strategy for ulterior patients. ‘Computational burden’ summarizes the computational resources consumed by the method, where No (Ø) refers to most resources consuming, Yes (^*^) refers to reasonably resources consuming and Very (^*^^*^^*^) refers to the best methods.

## DISCUSSION

In this work, we have selected four PM methods—MOM, BOSO, Lasso and KRL—and developed two additional ones—ODT and Multinomial Lasso—to compare them regarding their interpretability. We performed six quantitative comparisons and four qualitative comparisons. All the methods were similar in terms of accuracy. However, we suggest that MOM and ODT are the most interpretable and easy to implement.

PM is a topic that is being widely addressed and there are new algorithm proposals. It may seem surprising that we included only four of them in this comparison and, indeed, we developed two additional ones. A systematic review of all the methods—cited in the Introduction section—included ML methods (using deep learning, neural networks, support vector machines, random forests, etc.). Among the 24 methods that used ML for making their predictions, only 10 were explainable. Of those 10, 5 of them did not solve the ‘patient-centered’ problem: assign the proper drug to each patient. We ended up with MOM, BOSO, KRL and LOBICO, and added Lasso as a control of a traditional approach in the ML field. LOBICO approach, which was also tested on this dataset elsewhere [57], is drug-centered and, since the output variable is discrete, it cannot be transformed into a patient-centered problem and is not suitable for this comparison [57]. We developed two additional methods, both patient-centered, with two different approaches: regression (Multinomial) and tree classification (ODT).

In this work, we have defined interpretability, splitting it into four main concepts: accuracy, multi-omics capacity, explainability and implementability. An interpretable PM method should be accurate and understandable by the common researcher or clinician. Accuracy is strictly necessary: if a method is not accurate, it becomes irrelevant despite being easy to understand. Multi-omics capacity measures the ability of the method to accept different data source types, which could be essential for new lines of research. Explainability is also essential; it should show the reasoning for reaching the results. Finally, the ease of implementation defines the ability of the method to incorporate the clinician experience and provide easy technical usage.

We focused on a specific sensitivity value named IC_50_^*^. This metric was previously described in [54] or in [55] and is a normalization of the logarithm of the IC_50_. Normalizing the IC_50_—or other sensitivity value—is crucial as the best drug is not necessarily the drug with the lowest IC_50_ value. In fact, a drug with a low IC_50_ can be toxic for patients. Toxic drugs tend to have low IC_50_ values in all tissues, whereas the focus must be set on drugs with differential sensitivity for different tissues. Normalizing the logarithm of the IC_50_ by removing the mean sensitivity value of the drug in all patients preserves the sensitivity profiles of the drugs and penalizes drugs that are sensitive or resistant in all tissues. The dosage of drugs with higher IC_50_ can be adjusted to obtain drug effectiveness. We trained all the models with the normalized version, IC_50_^*^, to avoid the aforementioned problems.

The IC_50_^*^ improves the results with respect to the use of the standard IC_50_. For example, elesclomol, an extremely toxic compound, would be the indicated drug in many cases since its IC_50_ is very low [68]. On the other hand, the suggested treatments are reasonable according to the selected markers. Nevertheless, it is naive to think that using the IC_50_^*^ is ‘the’ solution to the PM problem. It is also necessary to consider the toxicity of each drug in different tissues, its pharmacodynamics, pharmacokinetics, ability to reach tumor cells with a therapeutic concentration, etc.

All the methods predict reasonably well in terms of accuracy. The 5-fold cross-validation, the test validation and the independent cohort validation showed that the different methods had similar medians and the differences were not statistically significant. The intragroup validation shows similar results: with the inherent problem of validating on cell lines instead of patient-derived cultivars.

The results of validation of cell lines are not as evident as those of patient-derived tissues. Cell lines are quite different from human tissues, and, in this case, the number of samples is much smaller. However, in most cases, the response to the proposed drug was better in the cell lines for which it was indicated than in those for which it was not.

The multi-omics suitability is a ‘hot topic’ in PM, as there is not a current gold standard based on which type of data is more accurate when predicting drug response. Some models use genetic variants to promote interpretability, whereas others use gene expression or integrated omics for improving accuracy. In this work, we compared the accuracy changes when training and predicting gene expression and genetic variants separately and found a small advantage for gene expression. Drug response is mediated in living beings by complex regulatory and metabolomic processes that are most likely to be solved using an integrated omics input, instead of just one single -omics. However, the more complex the model becomes, the less interpretable it is.

We also tested the models using both sources of data. In general, using both outperforms either of them but requires longer training time and more complex models.

Although different algorithms were tested using expression or expression together with mutations as input, we only tested the mutations approach on the GDSC dataset. In this case, gene expression was calculated using microarrays, and we did not even attempt to compare the results using RNAseq and microarrays. Mutations are much easier to compare between different datasets. Besides, mutations can be more reliably estimated from Formalin-Fixed Paraffin-Embedded (FFPE) samples than gene expression, as DNA is more stable than RNA and less susceptible to degradation caused by formalin fixation and long-term storage.

Metabolomics holds promise for PM, as it can distinguish between responders and non-responders to specific treatments [69]. Conceptually, the procedure would be identical to that using gene expression or mutational status, with the input being the concentration of various metabolites using, for example, LC/MS. Despite the BeatAML consortium plans to include metabolomics data, in this case, there are currently no metabolomics data available to test whether it outperforms other -omics. Nevertheless, it remains an interesting and promising avenue for research in this field.

Regarding explainability, we also included a qualitative comparison since focusing only on the number of variables does not justify that the method is understandable. It is also desirable that the method can provide decision criteria, i.e. a complete process that a clinician can follow and understand. This consideration has paramount importance if it is to be approved by regulators for medicine [70, 71]. Consequently, we focused on the ease to understand the output of the methods and the explainability of the results. We defined the latter, as the ability of the method to rank the input variables in order of importance for drug assignment. Of course, a smaller number of variables is easier to understand. The tree-based models require less than 10 variables, and the number increases in the regression-based models. BOSO, however, uses only five variables to predict the response of some drugs, but when translated into a patient-centered approach, the total number of variables used for predicting all drugs is equal to 49. For Lasso and Multinomial, the number of variables has been optimized to predict response in all drugs. KRL, however, did not consider this parameter and uses all variables provided as input to make the predictions, being the less explainable method.

Implementability is a concept easier to understand, as it directly facilitates the clinical translation. Most of the implementability comparisons were qualitative, but we performed a technical comparison of the methods regarding their computational burden. There we showed that MOM, which provides the simplest model with reasonable accuracy, requires the highest number of software environments: R, Python and CPLEX. The three of them must be installed on the machine and used together. It is quite resource consuming. If compared against ODT, which achieved better or similar accuracy, the latter only requires R, and the algorithm is trained, even using gene expression, in less than 5 s. On the other hand, Multinomial Lasso is also an accurate method.

Multinomial and Lasso are also explainable, although not as clear as decision trees. By a careful inspection of the *β* coefficients of the linear models (or its inclusion using Lasso penalties), it is possible to infer the importance of the variable for prediction.

We also believe that all methods could benefit from a graphical interface to help users understand their behavior and facilitate their use. However, ODT and MOM do not require it.

To summarize, in this work we defined a quantitative method for evaluating the interpretability of a given ML method because, as previously discussed, accuracy is not the only important factor in the complex field of health. The defined criteria can serve as a guide for developing new translational methods aimed at solving PM problems.

**Key points**

- For an ML method to be interpretable, it needs to be accurate, suitable to different multi-omics data, explainable and implementable.
- Traditional ML does not solve the complete assignment problem; thus, there are many creative methodologies to tackle drug assignment.
- There are several methods amenable to be interpretable and can be classified into two main groups: ‘patient-centered’ or ‘drug-centered’. ‘Drug-centered’ methods can be transformed into ‘patient-centered’ methods.
- In terms of explainability and implementability, the method needs to provide the decision criteria.
- From the methods compared—MOM, ODT, Multinomial, BOSO, KRL and Lasso—they all achieved similar results in terms of accuracy, ODT and MOM are the most explainable, and ODT the most implementable.

## Supplementary Material

Supplementary_Material_bbad200

## Data Availability

We selected the BeatAML2 cohort for training the models and predicting different therapeutic strategies. This cohort is publicly available at https://biodev.github.io/BeatAML2/. To validate the predictions, we used the GDSC drug screening for AML cell lines, which can be found publicly at https://www.cancerrxgene.org/. All the codebase required to execute all methods is available for download on GitHub (https://github.com/KatynaSada/InterpretableAIReview).

## Author Biographies

**Marian Gimeno** has a degree in Biomedical Engineering from the University of Navarra and a master's degree in Genomic Medicine from Imperial College London. She received her PhD from the University of Navarra.

**Katyna Sada del Real** is a Biomedical Engineer with a master's degree in Biomedical Data Analytics from the University of Navarra. She is currently pursuing her PhD in Artificial Intelligence for Cancer Research also at Tecnun University of Navarra.

**Angel Rubio** PhD is an Electrical Engineer from the Polytechnic University of Madrid. He received his PhD from the University of Navarra. He has been working in Computational Biology since 2001. His research interests are precision medicine, alternative splicing and translational bioinformatics.

## References

[1] Ashley EA . Towards precision medicine. Nat Rev Genet 2016;17:507–22. 27528417 10.1038/nrg.2016.86

[2] He M, Xia J, Shehab M, Wang X. The development of precision medicine in clinical practice. Clin Transl Med 2015;4:1. 26302883 10.1186/s40169-015-0069-y PMC4547974

[3] Collins FS, Varmus H. A new initiative on precision medicine. New England Journal of Medicine 2015;372:793–5. 25635347 10.1056/NEJMp1500523 PMC5101938

[4] Chen Y, Guzauskas GF, Gu C, et al. Precision health economics and outcomes research to support precision medicine: big data meets patient heterogeneity on the road to value. J Pers Med 2016;6(4):20. 10.3390/jpm6040020 PMC5198059 27827859

[5] Martorell-Marugán J, Tabik S, Benhammou Y, et al. Deep learning in omics data analysis and precision medicine. Computational Biology 2019;Chapter 3:37–53. 31815397

[6] González Burchard E, Borrell LN. Need for racial and ethnic diversity in asthma precision medicine. New England Journal of Medicine 2021;385:2297–8. 34879454 10.1056/NEJMe2114944

[7] Gerstung M, Papaemmanuil E, Martincorena I, et al. Precision oncology for acute myeloid leukemia using a knowledge bank approach. Nat Genet 2017;49:332–40. 28092685 10.1038/ng.3756 PMC5764082

[8] Xu J, Yang P, Xue S, et al. Translating cancer genomics into precision medicine with artificial intelligence: applications, challenges and future perspectives. Hum Genet 2019;138:109–24. 30671672 10.1007/s00439-019-01970-5 PMC6373233

[9] Li J, Zheng S, Chen B, et al. A survey of current trends in computational drug repositioning. Brief Bioinform 2016;17:2–12. 25832646 10.1093/bib/bbv020 PMC4719067

[10] Bhinder B, Gilvary C, Madhukar NS, Elemento O. Artificial intelligence in cancer research and precision medicine. Cancer Discov 2021;11:900–15. 33811123 10.1158/2159-8290.CD-21-0090 PMC8034385

[11] Stanfield Z, Coskun M, Koyutürk M. Drug response prediction as a link prediction problem. Sci Rep 2017;7:40321. 10.1038/srep40321 PMC5220354 28067293

[12] Wang L, Li X, Zhang L, Gao Q. Improved anticancer drug response prediction in cell lines using matrix factorization with similarity regularization. BMC Cancer 2017;17:513. 10.1186/s12885-017-3500-5 PMC5541434 28768489

[13] Su R, Liu X, Wei L, Zou Q. Deep-Resp-Forest: a deep forest model to predict anti-cancer drug response. Methods 2019;166:91–102. 30772464 10.1016/j.ymeth.2019.02.009

[14] Iorio F, Knijnenburg TA, Vis DJ, et al. A landscape of pharmacogenomic interactions in cancer. Cell 2016;166:740–54. 27397505 10.1016/j.cell.2016.06.017 PMC4967469

[15] Ma J, Yu MK, Fong S, et al. Using deep learning to model the hierarchical structure and function of a cell. Nat Methods 2018;15:290–8. 29505029 10.1038/nmeth.4627 PMC5882547

[16] Samal BR, Loers JU, Vermeirssen V, De Preter K. Opportunities and challenges in interpretable deep learning for drug sensitivity prediction of cancer cells. Frontiers in Bioinformatics 2022;2:2. 10.3389/fbinf.2022.1036963 PMC9714662 36466148

[17] Granat LM, Kambhampati O, Klosek S, et al. The promises and challenges of patient-derived tumor organoids in drug development and precision oncology. Animal Model Exp Med 2019;2:150–61. 31773090 10.1002/ame2.12077 PMC6762043

[18] Ben-David U, Ha G, Tseng YY, Greenwald NF, Oh C, Shih J, McFarland JM, Wong B, Boehm JS, Beroukhim R, Golub TR Patient-derived xenografts undergo murine-specific tumor evolution. Nat genet [internet]. NIH public Access; 2017 [cited 2023 Apr 4];49:1567. Available from: /pmc/articles/PMC5659952/, 75 10.1038/ng.3967 PMC5659952 28991255

[19] Roife D, Dai B, Kang Y, et al. Ex vivo testing of patient-derived xenografts mirrors the clinical outcome of patients with pancreatic ductal adenocarcinoma. Clin Cancer Res 2016;22:6021–30. 27259561 10.1158/1078-0432.CCR-15-2936 PMC5136340

[20] Shamout F, Zhu T, Clifton DA. Machine learning for clinical outcome prediction. IEEE Rev Biomed Eng 2021;14:116–26. 32746368 10.1109/RBME.2020.3007816

[21] Scott IA, Cook D, Coiera EW, Richards B. Machine learning in clinical practice: prospects and pitfalls. Medical Journal of Australia 2019;211:203. 31389031 10.5694/mja2.50294

[22] Adlung L, Cohen Y, Mor U, Elinav E. Machine learning in clinical decision making. Med 2021;2:642–65. 35590138 10.1016/j.medj.2021.04.006

[23] Oh SH, Lee SJ, Park J. Precision medicine for hypertension patients with type 2 diabetes via reinforcement learning. Journal of Personalized Medicine 2022;12:87. 35055402 10.3390/jpm12010087 PMC8781402

[24] Eckardt J-N, Wendt K, Bornhäuser M, et al. Reinforcement learning for precision oncology. Cancer 2021;13(18):4624. 10.3390/cancers13184624 PMC8472712 34572853

[25] Liu Q, Hu Z, Jiang R, Zhou M. DeepCDR: a hybrid graph convolutional network for predicting cancer drug response. Bioinformatics 2020;36:i911–8. 33381841 10.1093/bioinformatics/btaa822

[26] Lee BKB, Tiong KH, Chang JK, et al. DeSigN: connecting gene expression with therapeutics for drug repurposing and development. BMC Genomics 2017;18:934. 28198666 10.1186/s12864-016-3260-7 PMC5310278

[27] Preuer K, Lewis RPI, Hochreiter S, et al. DeepSynergy: predicting anti-cancer drug synergy with deep learning. Wren J, editor. Bioinformatics 2018;34:1538–46. 29253077 10.1093/bioinformatics/btx806 PMC5925774

[28] Robert J, Vekris A, Pourquier P, Bonnet J. Predicting drug response based on gene expression. Crit Rev Oncol Hematol 2004;3:205–27. 10.1016/j.critrevonc.2004.06.002 15331079

[29] Seo H, Tkachuk D, Ho C, et al. SYNERGxDB: an integrative pharmacogenomic portal to identify synergistic drug combinations for precision oncology. Nucleic Acids Res 2020;48:W494–501. 32442307 10.1093/nar/gkaa421 PMC7319572

[30] Lind AP, Anderson PC. Predicting drug activity against cancer cells by random forest models based on minimal genomic information and chemical properties. Olier I, editor. PloS One 2019;14:e0219774. 31295321 10.1371/journal.pone.0219774 PMC6622537

[31] Boichard A, Richard SB, Kurzrock R. The crossroads of precision medicine and therapeutic decision-making: use of an analytical computational platform to predict response to cancer treatments. Cancers (Basel) 2020;12:166. 31936627 10.3390/cancers12010166 PMC7017109

[32] Siah KW, Khozin S, Wong CH, Lo AW. Machine-learning and stochastic tumor growth models for predicting outcomes in patients with advanced non–small-cell lung cancer. JCO Clin. Cancer Inform 2019;3:1–11. 10.1200/CCI.19.00046 31539267

[33] Chang Y, Park H, Yang HJ, et al. Cancer drug response profile scan (CDRscan): a deep learning model that predicts drug effectiveness from cancer genomic signature. Sci Rep 2018;8. 10.1038/s41598-018-27214-6 PMC5996063 29891981

[34] Joo M, Park A, Kim K, et al. A deep learning model for cell growth inhibition IC50 prediction and its application for gastric cancer patients. Int J Mol Sci 2019;20:6276. 31842404 10.3390/ijms20246276 PMC6941066

[35] Huang C, Clayton EA, Matyunina LV, et al. Machine learning predicts individual cancer patient responses to therapeutic drugs with high accuracy. Sci Rep 2018;8:8. 30401894 10.1038/s41598-018-34753-5 PMC6219522

[36] Guo W, Ji Y, Catenacci DVT. A subgroup cluster-based Bayesian adaptive design for precision medicine. Biometrics 2017;73:367–77. 27775814 10.1111/biom.12613 PMC5923898

[37] Kim Y, Kim D, Cao B, et al. PDXGEM: patient-derived tumor xenograft-based gene expression model for predicting clinical response to anticancer therapy in cancer patients. BMC Bioinformatics 2020;21:288. 32631229 10.1186/s12859-020-03633-z PMC7336455

[38] Matchett K, Lynam-Lennon N, Watson R, Brown J. Advances in precision medicine: tailoring individualized therapies. Cancers (Basel) 2017;9:146. 29068364 10.3390/cancers9110146 PMC5704164

[39] Azuaje F . Artificial intelligence for precision oncology: beyond patient stratification. NPJ Precis Oncol 2019;3:1–5. 30820462 10.1038/s41698-019-0078-1 PMC6389974

[40] Biankin AV . The road to precision oncology. Nat Genet 2017;49:320–1. 28232728 10.1038/ng.3796

[41] Chua IS, Gaziel‐Yablowitz M, Korach ZT, et al. Artificial intelligence in oncology: path to implementation. Cancer Med 2021;10:4138–49. 33960708 10.1002/cam4.3935 PMC8209596

[42] Jiménez-Luna J, Grisoni F, Schneider G. Drug discovery with explainable artificial intelligence. Nat Mach Intell 2020;2:573–84.

[43] Kuenzi BM, Park J, Fong SH, et al. Predicting drug response and synergy using a deep learning model of human cancer cells. Cancer Cell 2020;38:672–684.e6. 33096023 10.1016/j.ccell.2020.09.014 PMC7737474

[44] Khakabimamaghani S, Kelkar YD, Grande BM, et al. SUBSTRA: supervised Bayesian patient stratification. Berger B, editor. Bioinformatics 2019;35:3263–72. 30768166 10.1093/bioinformatics/btz112

[45] Kim Y, Bismeijer T, Zwart W, et al. Genomic data integration by WON-PARAFAC identifies interpretable factors for predicting drug-sensitivity in vivo. Nat Commun 2019;10:1–12. 31695042 10.1038/s41467-019-13027-2 PMC6834616

[46] Vougas K, Sakellaropoulos T, Kotsinas A, et al. Machine learning and data mining frameworks for predicting drug response in cancer: an overview and a novel in silico screening process based on association rule mining. Pharmacol Ther 2019;203:107395. 31374225 10.1016/j.pharmthera.2019.107395

[47] Astras G, Papagiannopoulos CI, Kyritsis KA, et al. Pharmacogenomic testing to guide personalized cancer medicine decisions in private oncology practice: a case study. Front Oncol 2020;10:521. 32411592 10.3389/fonc.2020.00521 PMC7199631

[48] Pai S, Hui S, Isserlin R, et al. netDx: interpretable patient classification using integrated patient similarity networks. Mol Syst Biol 2019;15:e8497. 30872331 10.15252/msb.20188497 PMC6423721

[49] Liu H, Zhao R, Fang H, et al. Entropy-based consensus clustering for patient stratification. Bar-Joseph Z, editor. Bioinformatics 2017;33:2691–8. 28369256 10.1093/bioinformatics/btx167

[50] Stetson LC, Pearl T, Chen Y, Barnholtz-Sloan JS. Computational identification of multi-omic correlates of anticancer therapeutic response. BMC Genomics 2014;15:S2. 10.1186/1471-2164-15-S7-S2 PMC4243102 25573145

[51] Ingelman-Sundberg M, Mkrtchian S, Zhou Y, Lauschke VM. Integrating rare genetic variants into pharmacogenetic drug response predictions. Hum Genomics 2018;12:26. 29793534 10.1186/s40246-018-0157-3 PMC5968569

[52] Oberthuer A, Juraeva D, Hero B, et al. Revised risk estimation and treatment stratification of low- and intermediate-risk neuroblastoma patients by integrating clinical and molecular prognostic markers. Clin Cancer Res 2015;21:1904–15. 25231397 10.1158/1078-0432.CCR-14-0817

[53] Cheng L, Majumdar A, Stover D, et al. Computational cancer cell models to guide precision breast cancer medicine. Genes (Basel) 2020;11:263. 32121160 10.3390/genes11030263 PMC7140855

[54] Gimeno M, San José-Enériz E, Villar Fernandez S, et al. Explainable artificial intelligence for precision medicine in acute myeloid leukemia. Front Immunol 2022;13:5805. 10.3389/fimmu.2022.977358 PMC9556772 36248800

[55] He X, Folkman L, Borgwardt K. Kernelized rank learning for personalized drug recommendation. Wren J, editor. Bioinformatics 2018;34:2808–16. 29528376 10.1093/bioinformatics/bty132 PMC6084606

[56] Valcárcel LV, San José-Enériz E, Cendoya X, et al. BOSO: a novel feature selection algorithm for linear regression with high-dimensional data. Kosakovsky Pond SL, editor. PLoS Comput Biol 2022;18:e1010180. 35639775 10.1371/journal.pcbi.1010180 PMC9187084

[57] Knijnenburg TA, Klau GW, Iorio F, et al. Logic models to predict continuous outputs based on binary inputs with an application to personalized cancer therapy. Sci Rep 2016;6:1–14. 27876821 10.1038/srep36812 PMC5120272

[58] Friedman J, Hastie T, Tibshirani R. Regularization paths for generalized linear models via coordinate descent. J Stat Softw 2010;33:1–22. 20808728 PMC2929880

[59] Tyner JW, Tognon CE, Bottomly D, et al. Functional genomic landscape of acute myeloid leukaemia. Nature 2018;562:526–31. 30333627 10.1038/s41586-018-0623-z PMC6280667

[60] Yang W, Soares J, Greninger P, et al. Genomics of drug sensitivity in cancer (GDSC): a resource for therapeutic biomarker discovery in cancer cells. Nucleic Acids Res 2012;41:D955–61. 23180760 10.1093/nar/gks1111 PMC3531057

[61] Zeisig BB, Kulasekararaj AG, Mufti GJ, Eric So CW. SnapShot: acute myeloid leukemia. Cancer Cell 2012;22:698–698.e1. 23153541 10.1016/j.ccr.2012.10.017

[62] Döhner H, Estey E, Grimwade D, et al. Diagnosis and management of AML in adults: 2017 ELN recommendations from an international expert panel. Blood 2017;129:424–47. 27895058 10.1182/blood-2016-08-733196 PMC5291965

[63] NIH NCInstituteGDC . Acute Myeloid Leukemia — Cancer Stat Facts.

[64] Döhner H, Estey EH, Amadori S, et al. Diagnosis and management of acute myeloid leukemia in adults: recommendations from an international expert panel, on behalf of the European LeukemiaNet. Blood 2010;115:453–74. 19880497 10.1182/blood-2009-07-235358

[65] Kucukyurt S, Eskazan AE. New drugs approved for acute myeloid leukaemia in 2018. Br J Clin Pharmacol [Internet]. Wiley-Blackwell; 2019 [cited 2023 Apr 4];85:2689. Available from: /pmc/articles/PMC6955409/31469910 10.1111/bcp.14105 PMC6955409

[66] Ahmad MA, Eckert C, Teredesai A. Interpretable machine learning in healthcare*. Proceedings of the 2018 ACM International Conference on Bioinformatics, Computational Biology, and Health Informatics*. New York, NY, USA: ACM, 2018, 559–60.

[67] Lucena-Araujo AR, Coelho-Silva JL, Pereira-Martins DA, et al. Combining gene mutation with gene expression analysis improves outcome prediction in acute promyelocytic leukemia. Blood 2019;134:951–9. 31292112 10.1182/blood.2019000239 PMC7484742

[68] Gimeno M, Gimeno M, San José -Ené Riz E, et al. Explainable artificial intelligence for precision medicine in acute myeloid leukemia. Front Immunol. 2022;13. 10.3389/fimmu.2022.977358 PMC9556772 36248800

[69] Wishart DS . Emerging applications of metabolomics in drug discovery and precision medicine. Nat Rev Drug Discov 2016;15:473–84. 26965202 10.1038/nrd.2016.32

[70] Artificial intelligence in medicine regulation | European Medicines Agency [Internet]. https://www.ema.europa.eu/en/news/artificial-intelligence-medicine-regulation (cited 15 March 2022).

[71] U.S. Food and Drug Administration . Proposed regulatory framework for modifications to artificial intelligence/machine learning (AI/ML)-based software as a medical device (SaMD)-discussion paper and request for feedback. FDA 2019;20. https://www.fda.gov/files/medicaldevices/published/US-FDA-Artificial-Intelligence-and-Machine-Learning-Discussion-Paper.pdf.

