# Supplementary material for: Precision oncology: a review to assess interpretability in several explainable methods
Source: Brief Bioinform. 2023 May 30;24(4):bbad200. doi: 10.1093/bib/bbad200 (PMC10359088; doi:10.1093/bib/bbad200)
Supplement: Supplementary_Material_bbad200 [file supplementary_material_bbad200.docx]

*Supplementary*: Precision Oncology: a review to assess interpretability in several explainable methods

Marian Gimeno^1^, Katyna Sada^1^ and Angel Rubio^1,2^*

^1^ Departamento de Ingeniería Biomédica y Ciencias, TECNUN, Universidad de Navarra, 20009, San Sebastián, Spain.

^2^ Instituto de Ciencia de los Datos e Inteligencia Artificial (DATAI), Universidad de Navarra, 31008, Pamplona, Spain.

**Corresponding Author**

Angel Rubio

Tel.:+34 943 21 98 77; E-mail: arubio@tecnun.es

# Supplementary Methods

## Multi-dimensional Optimization Module (MOM)

Multidimensional Optimization Module (MOM) is a novel method that was built up previously by this group which intends to give a highly clinical translational therapeutic strategy [1].

The method is defined by three steps, i) normalizing IC_50_ value (IC50*) to reduce potential drug toxicity, ii) selecting possible relevant cohort biomarkers using HUGE methodology [2], and iii) optimize the treatment strategy using a MILP model that joins the normalized drug response values and effective biomarkers to output an effective treatment strategy.

This model finds the optimal distribution for the subgroups according to the maximum patient response. The MILP model receives as input an X binary matrix that contains the biomarkers (genetic variants or gene fusions) that are present in each of the patients, a Y matrix containing the IC50* value for each patient and each of the 122 drugs in the BeatAML cohort [3], and a binary matrix that relates the biomarkers to the drugs, meaning the biomarkers that potentially suggest drug sensitivity for a certain treatment. The step parameter was set to 4, meaning that the model will output four different subgroups.

We implemented the model in the manner that it was mentioned in its publication, with the same training cohort and parameters. Model validation was performed in Genomics of Drug Senstivity in Cancer (GDSC) AML cohort [4].

## Lasso regression

The assignation problem can be divided into two steps: the prediction of the effectiveness of each drug on each patient and, afterward, the selection of he most effective drug according to the predictions. The first step is a standard regression problem that can be solved using any technique amenable for regression: linear regression, SVM as well as almost any supervised machine learning method. The second step is simply the selection of the most effective drug for each patient.

Since the number of regressors (genetic characteristics) is much larger than the number of samples (number of patients), it is advisable to include some type of regularization to prevent overfitting as done using the Lasso or the elastic net regression. In mathematical terms the aim is to find the set of regressors $\boldsymbol{\beta}$ so that, the predicted response is similar to the actual response (**Equation 7**).

| $\boldsymbol{X\beta}\sim\boldsymbol{Y}$ | (7) |
| --- | --- |

where **X** is a P x M matrix where P is the number of patients and M is the number of biomarkers, $\boldsymbol{Y}$ is P x D matrix of effectiveness where P is the number of patients and D is the number of tested drugs. Finally$\boldsymbol{\beta}$**,** the output of the regression is a M x D coefficient matrix. $\boldsymbol{X\beta}$ are the predicted effectivities for each. Computing the minimum value of $\boldsymbol{X\beta}$ estimates the most effective drug for each patient.

## BOSO

The application of BOSO is conceptually similar to Lasso: a regression model is built and BOSO, using mixed integer programming techniques, estimates the regressor matrix.

BOSO is very efficient finding a minimum number of parameters. In fact, for the simulated dataset of the reference, BOSO outperforms Lasso in any condition for the same number of not-null coefficients in the regressor [5].

## Kernelized Rank Learning (KRL)

Kernelized Rank Learning is a novel methodology developed by He, X. *et al.* [6] that recommends a drug to a specific patient. This methodology is computed using a regression method for which by using a kernel to the input data and applying a regularization term that strongly depends on the ranking of the patient’s sensitivity to a certain drug, by minimizing the error, it outputs the specific patient recommendation.

## References

1. Gimeno M, San José-Enériz E, Villar Fernandez S, et al. Explainable Artificial Intelligence for Precision Medicine in Acute Myeloid Leukemia. Front. Immunol. 2022; 0:5805

2. Gimeno M, San José-Enériz E, Rubio A, et al. Identifying Lethal Dependencies with HUGE Predictive Power. Cancers (Basel). 2022; 14:3251

3. Tyner JW, Tognon CE, Bottomly D, et al. Functional genomic landscape of acute myeloid leukaemia. Nature 2018; 562:526–531

4. Yang W, Soares J, Greninger P, et al. Genomics of Drug Sensitivity in Cancer (GDSC): a resource for therapeutic biomarker discovery in cancer cells. Nucleic Acids Res. 2012; 41:D955–D961

5. Valcárcel L V., San José-Enériz E, Cendoya X, et al. BOSO: A novel feature selection algorithm for linear regression with high-dimensional data. PLOS Comput. Biol. 2022; 18:e1010180

6. He X, Folkman L, Borgwardt K. Kernelized rank learning for personalized drug recommendation. Bioinformatics 2018; 34:2808–2816

# Supplementary Figures

## Supplementary Figure 1: Violin plots showing distribution in 5-fold cross validation.


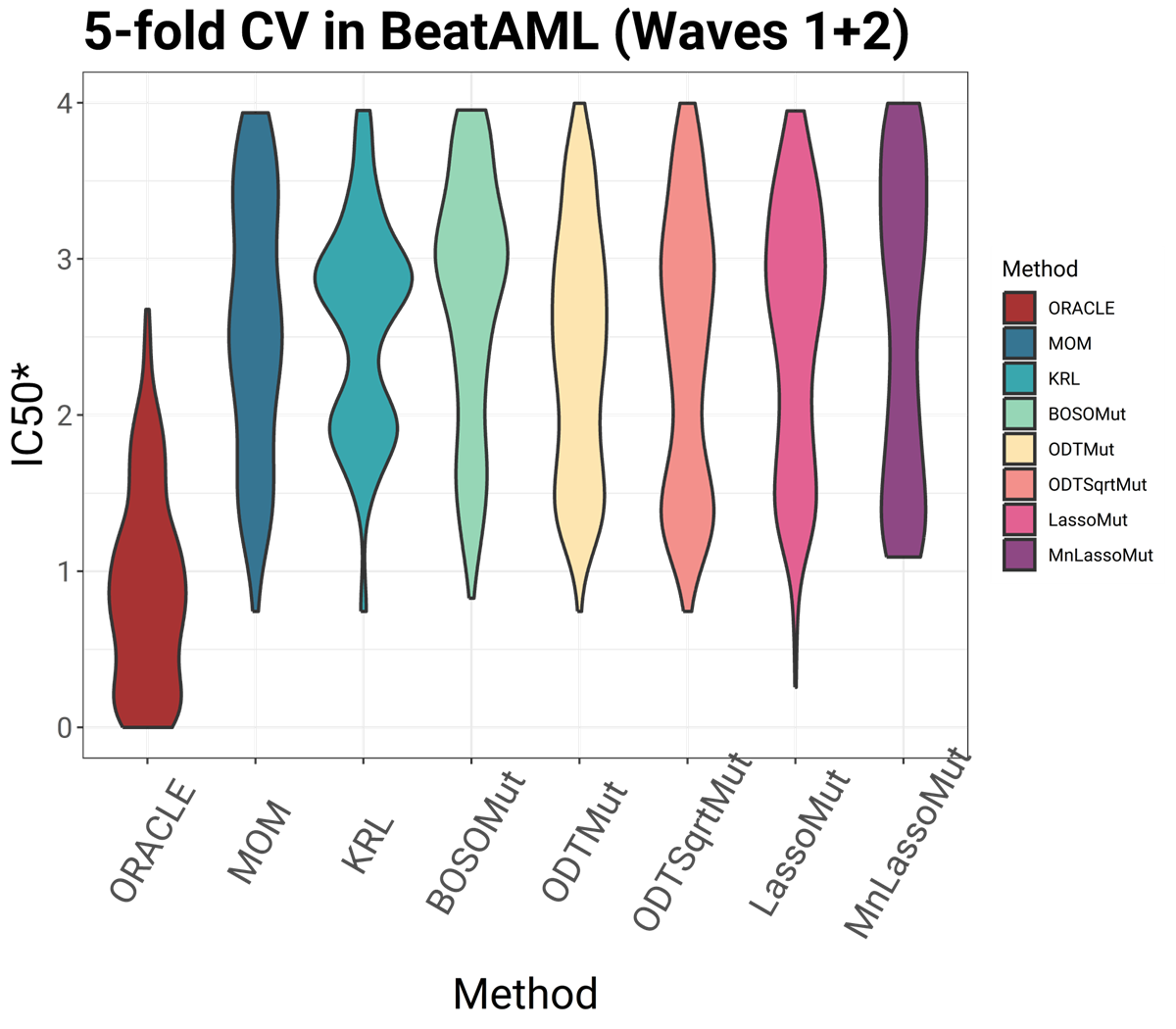


## Supplementary Figure 2: Violin plots showing distribution in GDSC validation.


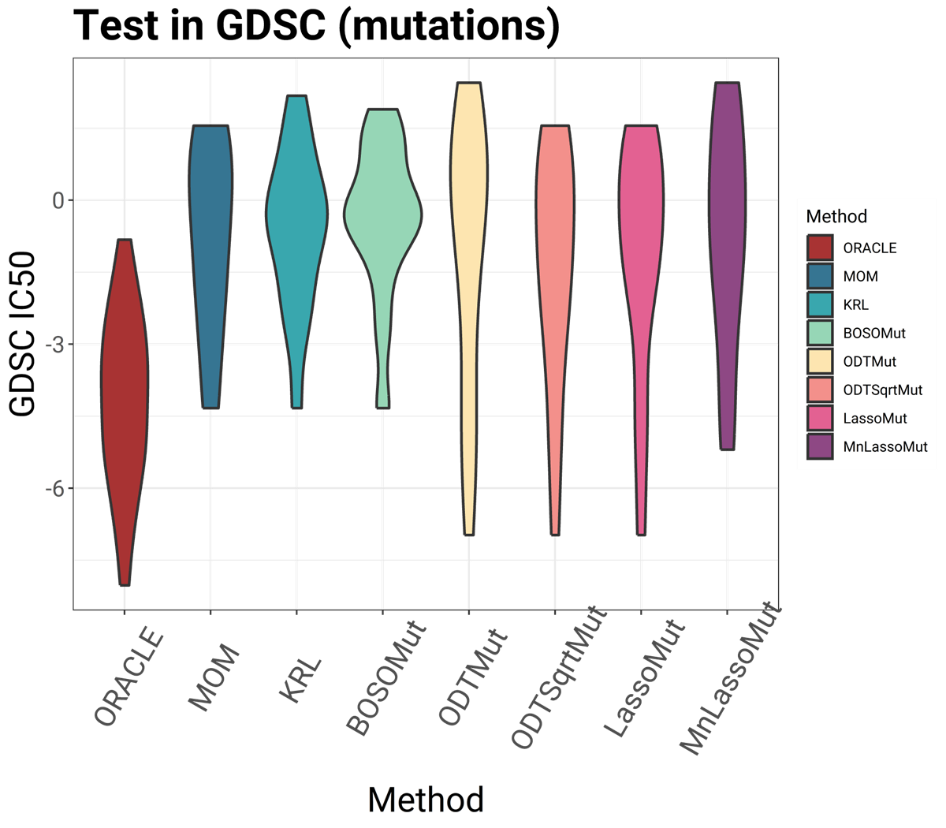


## Supplementary Figure 3: Validation in DEMETER2 and CERES.


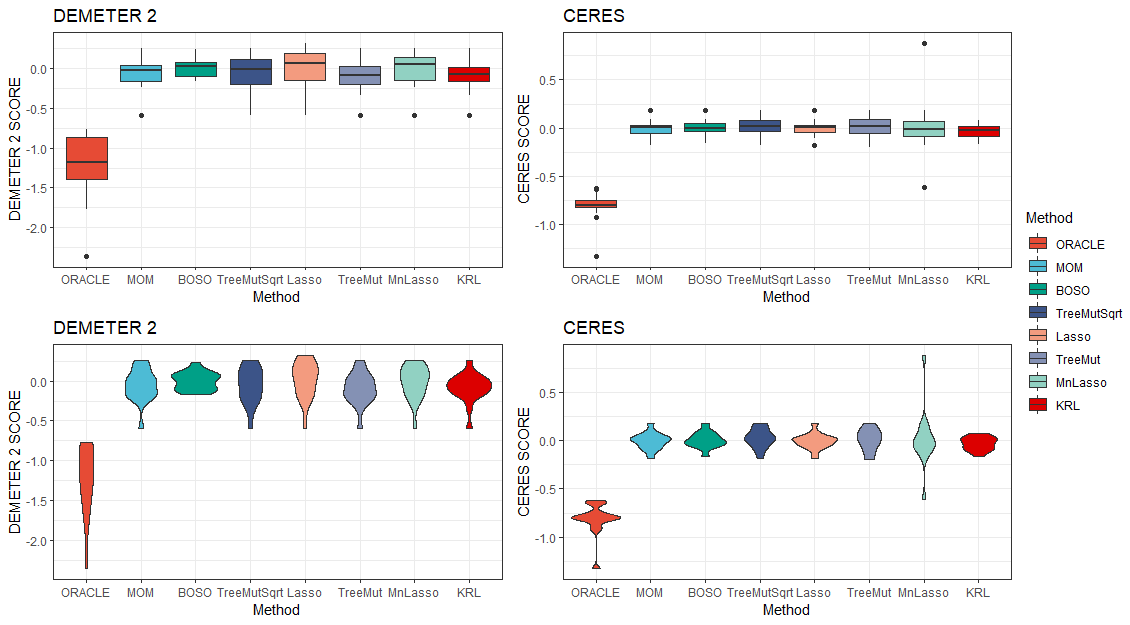


## Supplementary Figure 4: Statistical significance between the different therapeutic strategies using BOSO in BeatAML Waves 3+4.


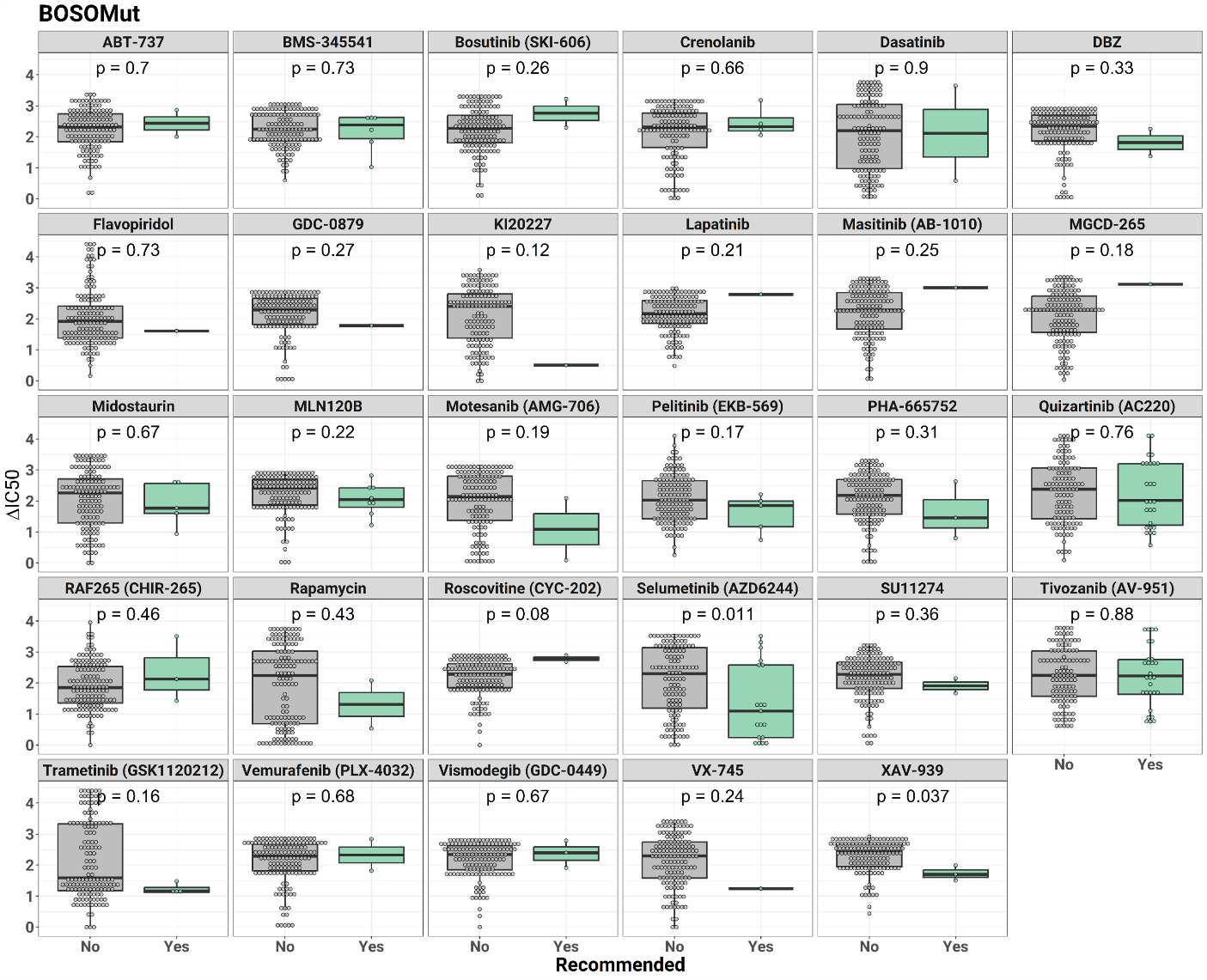


## Supplementary Figure 5: Statistical significance between the different therapeutic strategies using BOSO in GDSC.


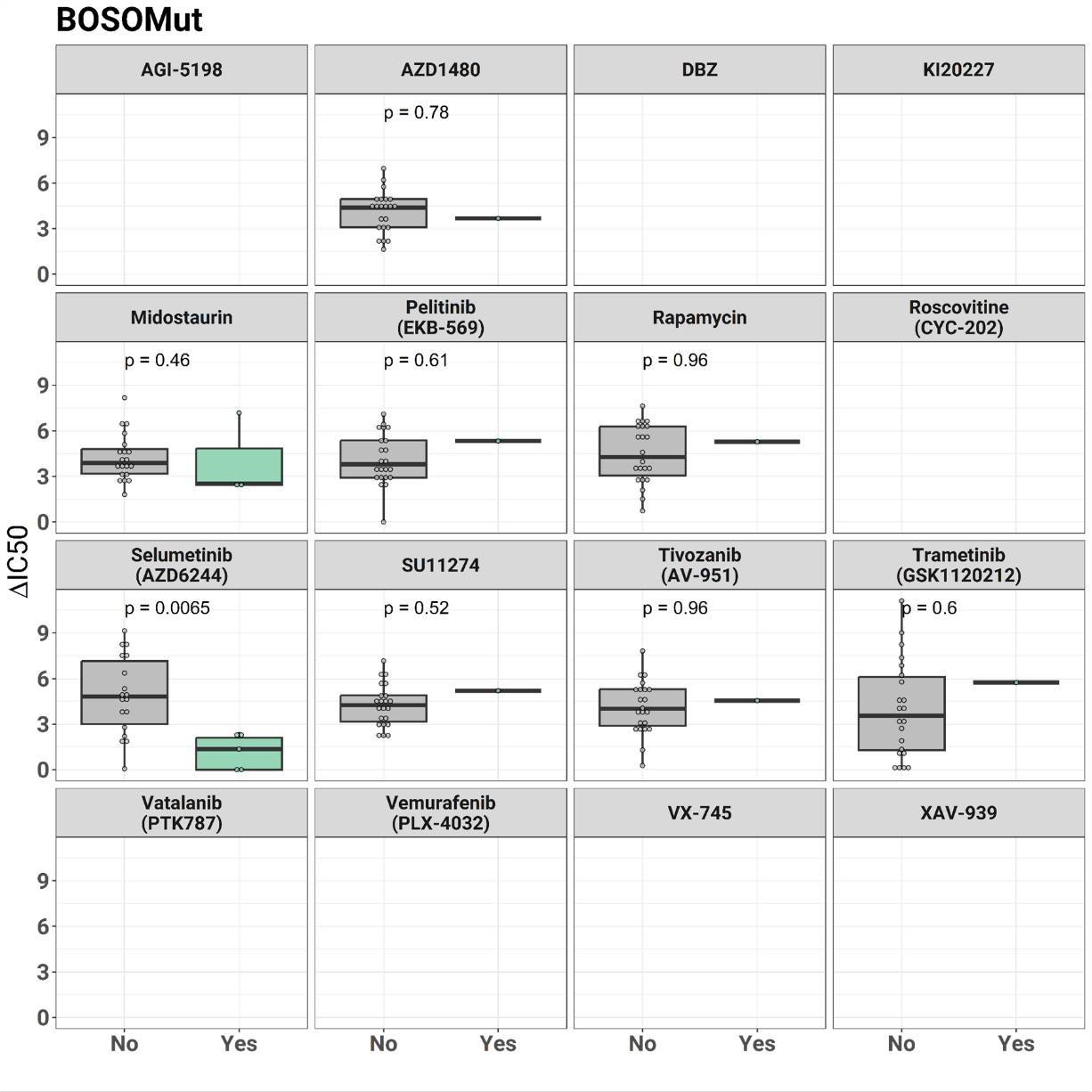


## Supplementary Figure 6: Statistical significance between the different therapeutic strategies using Lasso in BeatAML.


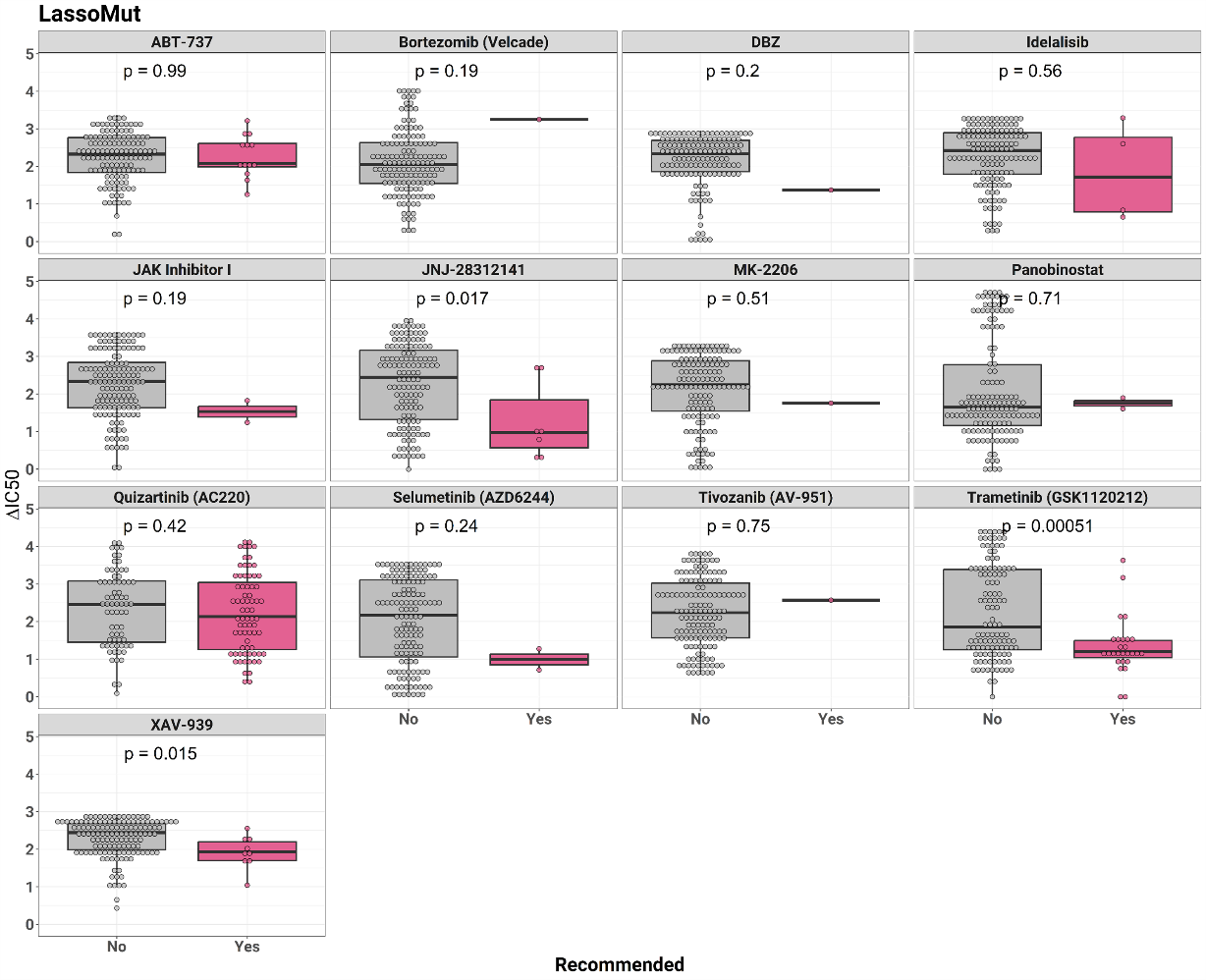


## Supplementary Figure 7: Statistical significance between the different therapeutic strategies using Lasso in GDSC.


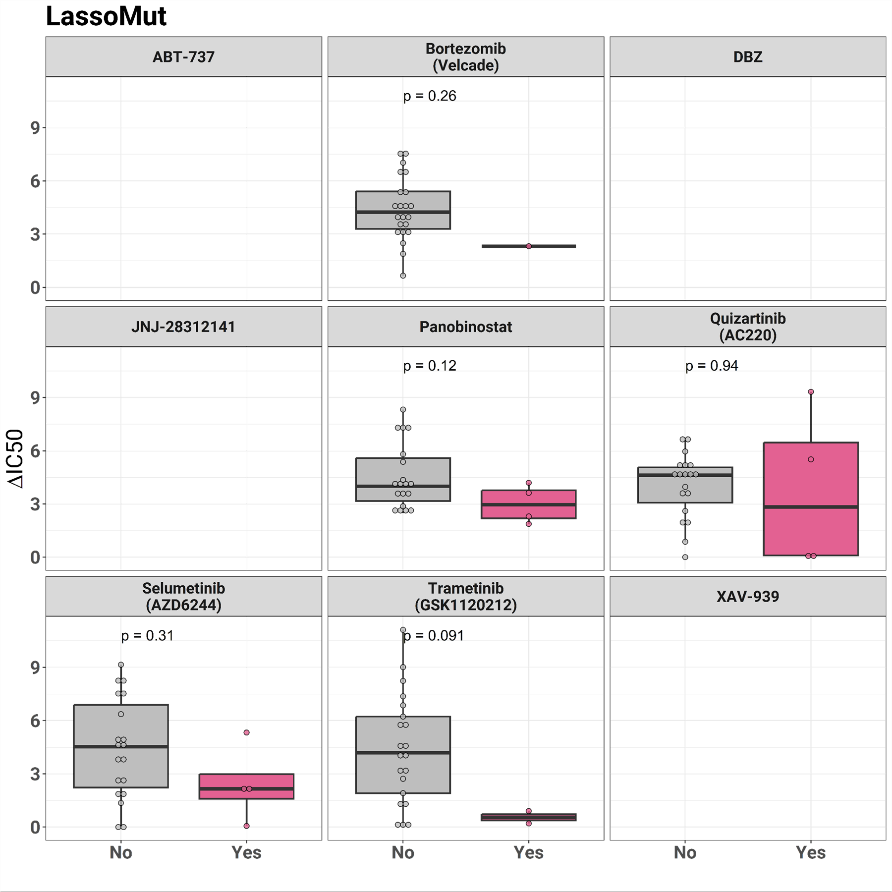


## Supplementary Figure 8: Statistical significance between the different therapeutic strategies using Multinomial in BeatAML.


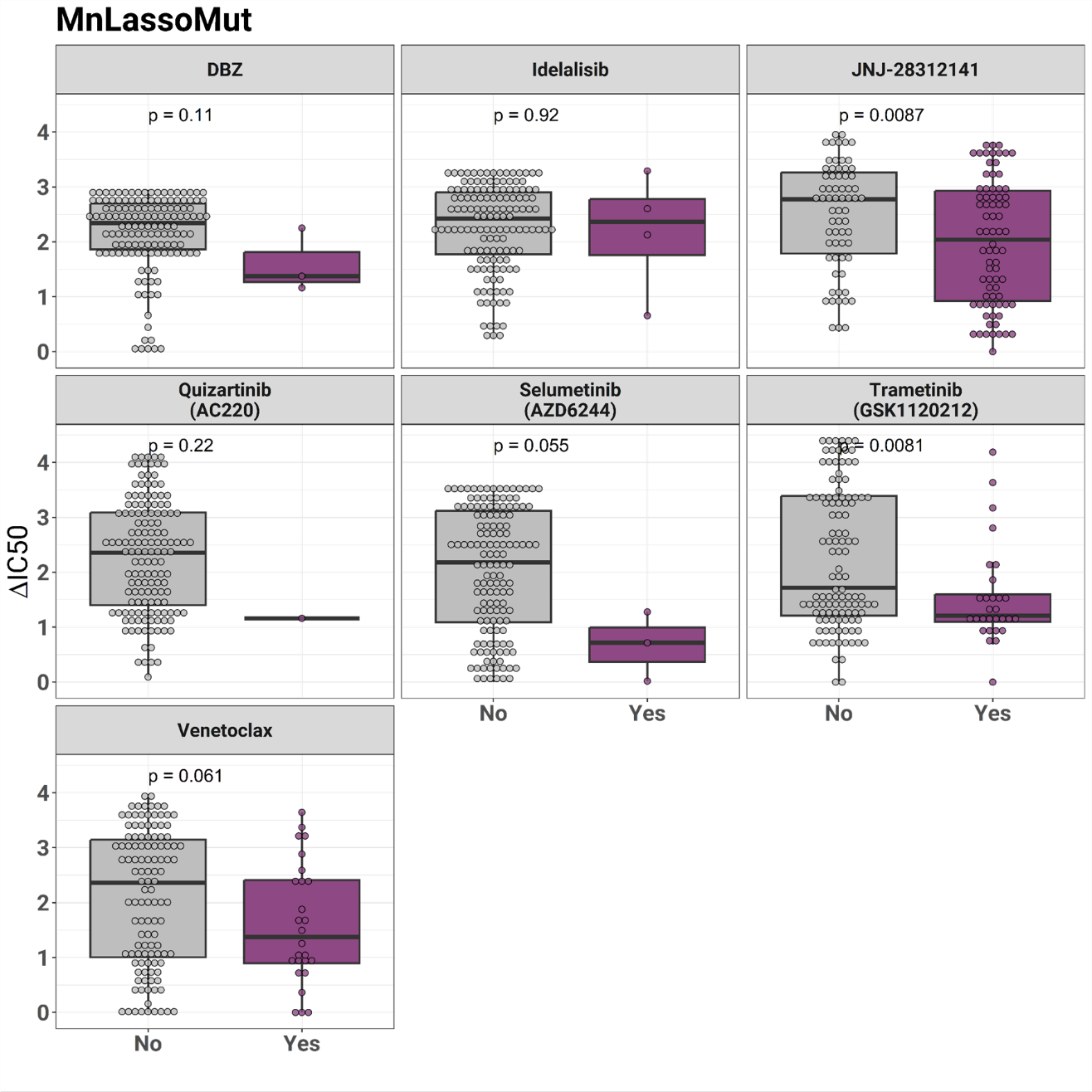


## Supplementary Figure 9: Statistical significance between the different therapeutic strategies using Multinomial in GDSC.


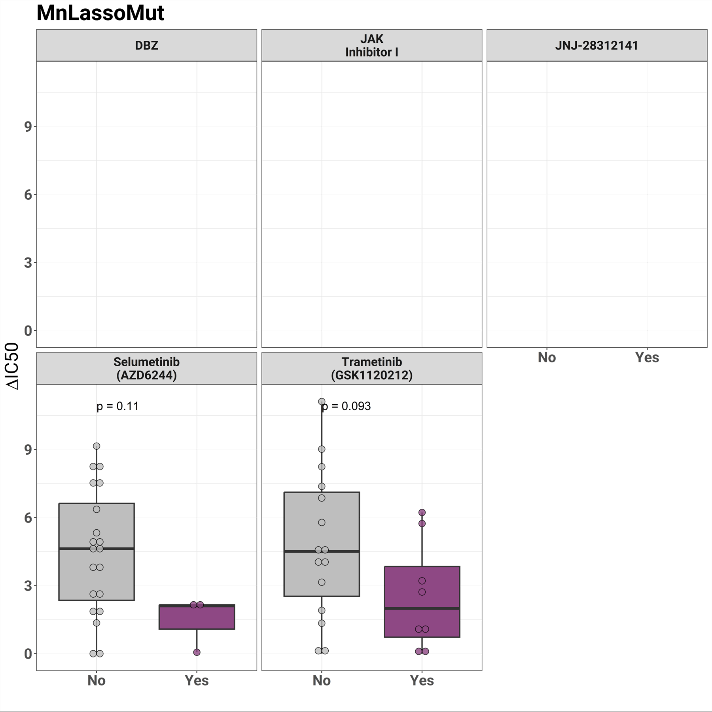


## Supplementary Figure 10: Statistical significance between the different therapeutic strategies using KRL in BeatAML.


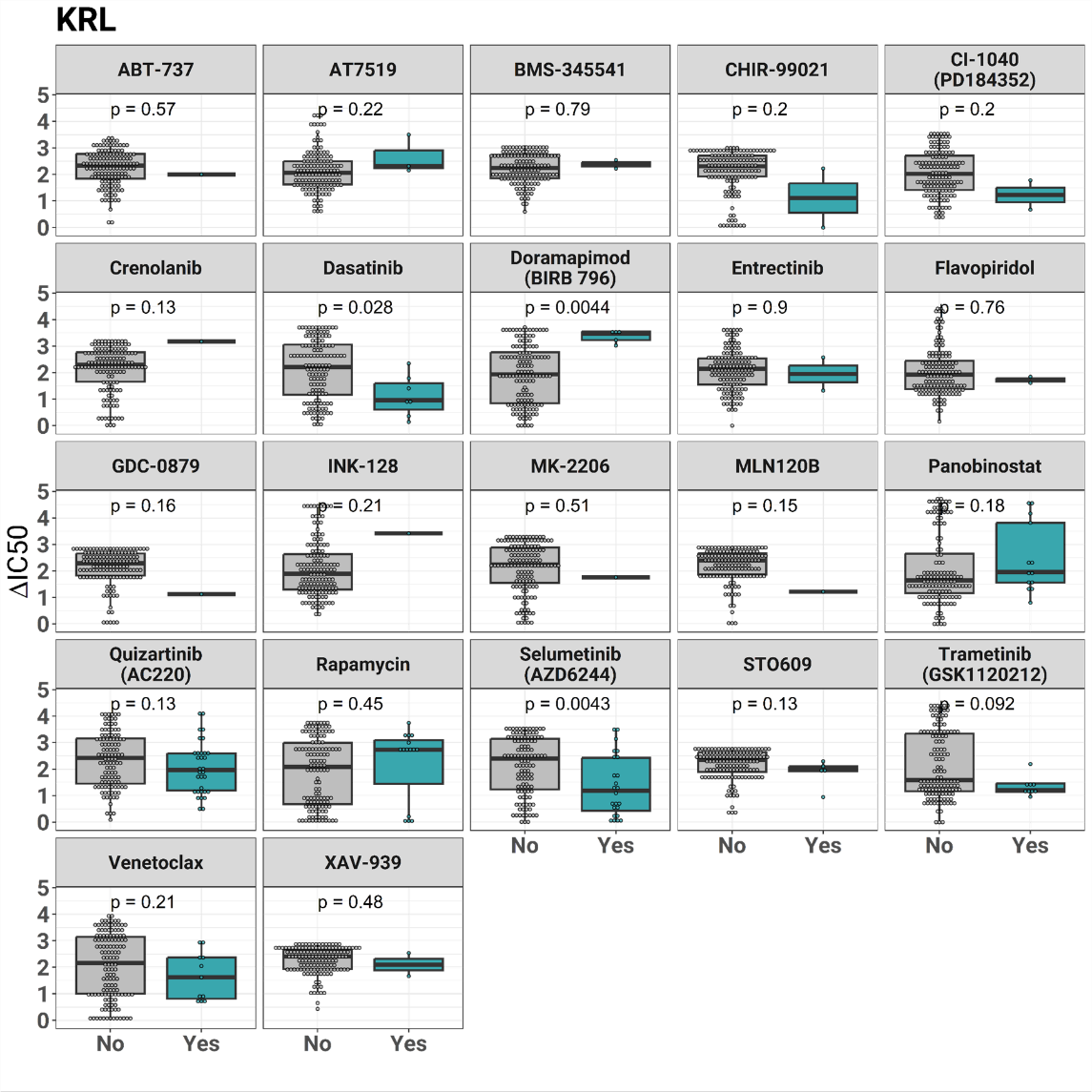


## Supplementary Figure 11: Statistical significance between the different therapeutic strategies using KRL in GDSC.


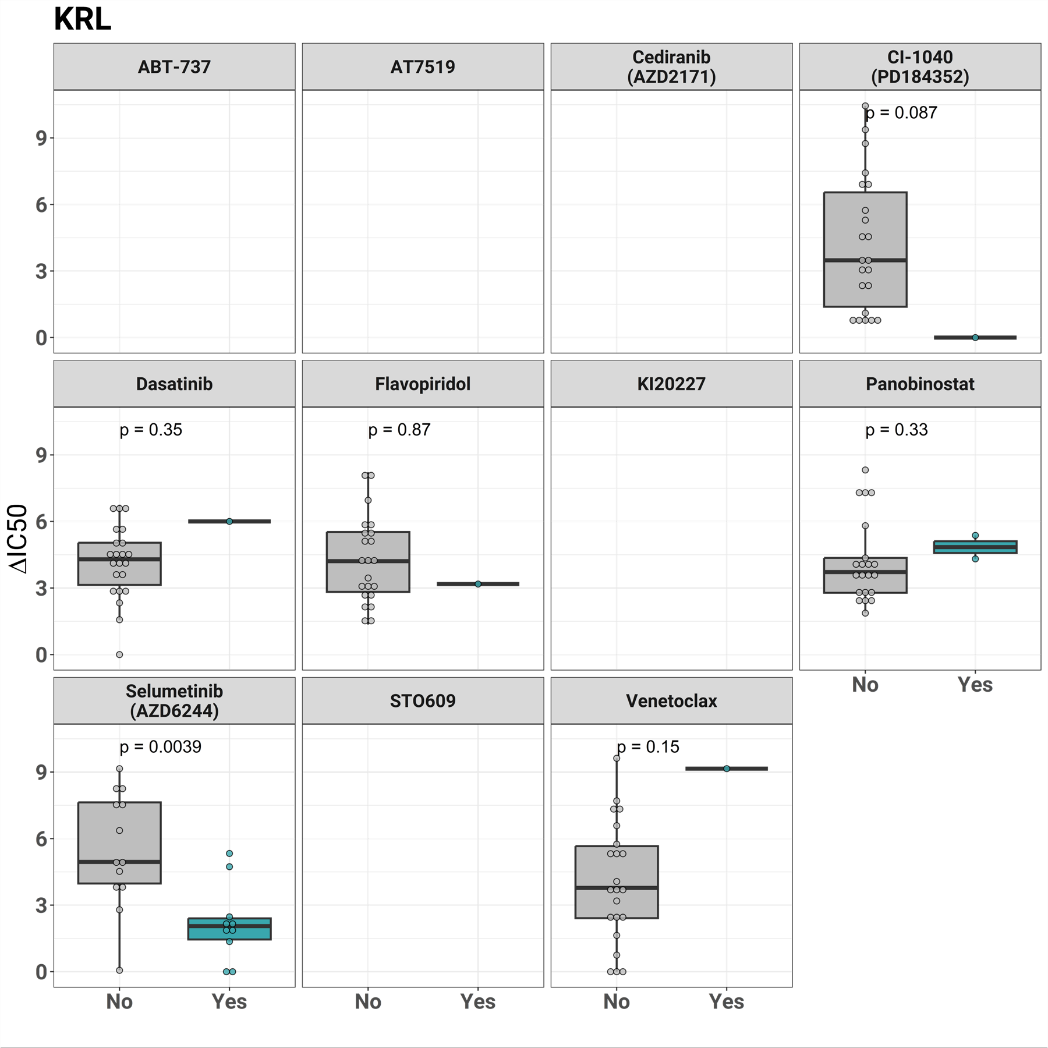


## Supplementary Figure 12: Lasso Mut Variables


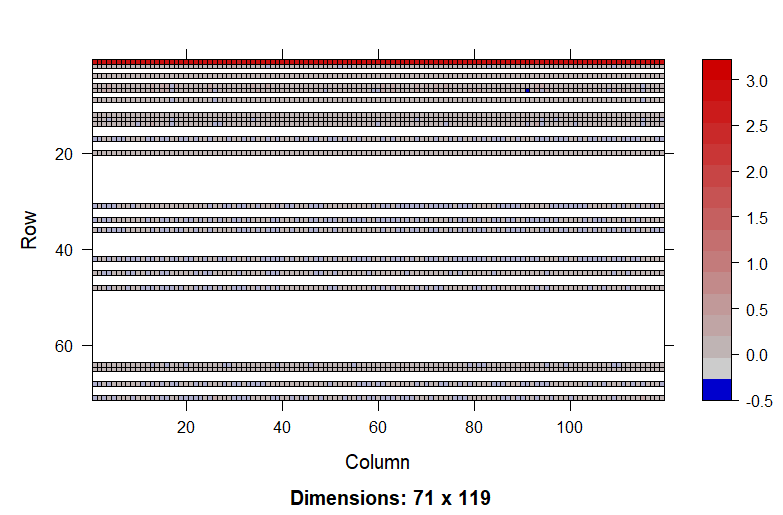


## Supplementary Figure 13: MnLasso Mut Variables


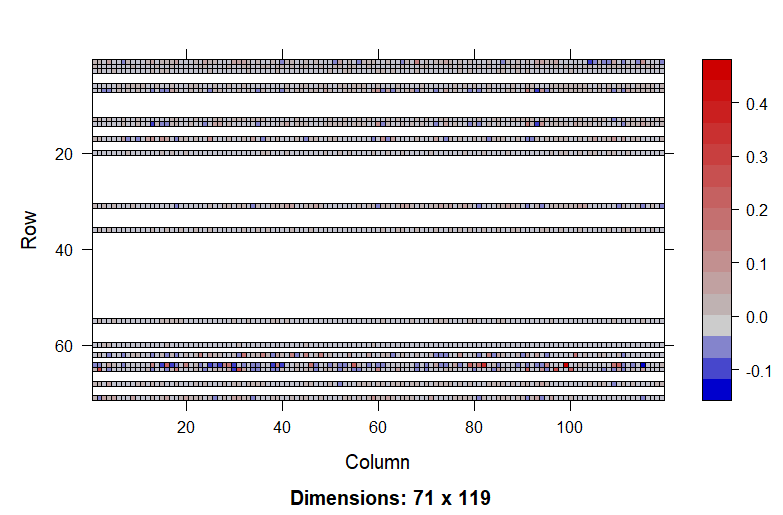


## Supplementary Figure 14: BOSO Mut variables


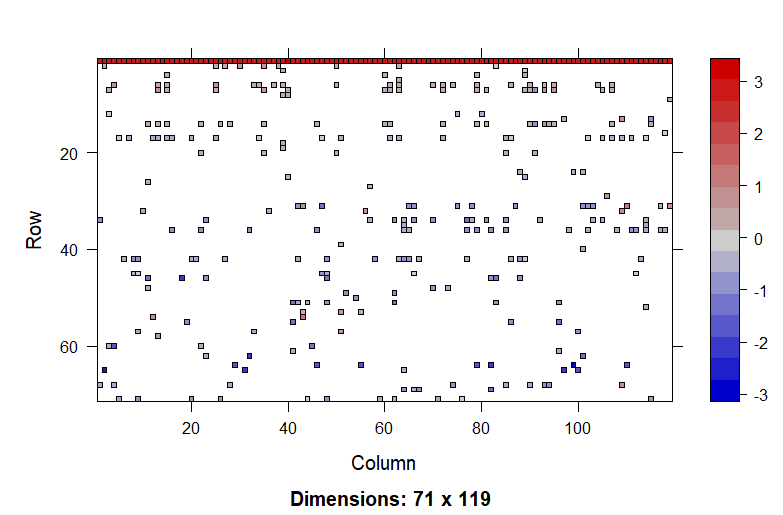


| **Dataset** | **Number of samples** | **Number of samples with gene expression, mutations and drug response** | **Number of drugs tested in at least 30% of samples** | **Treatment types** |
| --- | --- | --- | --- | --- |
| BeatAML waves 1+2 | 518 | 232 | 119 | Standard Chemotherapy, Targeted Therapy, Bone Marrow Transplant, Intrathecal, Supportive/Palliative Care |
| BeatAML waves 3+4 | 228 | 142 | 119 |  |
| BeatAML Both | 15 | 15 | 119 |  |
| GDSC AML samples | 23 | 23 | 27 in common with BeatAML (54 in total) |  |

Supplementary Table 1 Information on data sets
